# Adipocyte OX40L promotes adipose T cell activation and insulin resistance in obesity

**DOI:** 10.1038/s12276-026-01770-8

**Published:** 2026-07-06

**Authors:** Jianfeng Song, Qin Zeng, Yayi Jiao, Xiaoxiao Sun, Limin Xie, Jingpei Qiu, Yujin Ding, Wanyu Hu, Fanqi Wang, Biling Huang, Wuqian Mai, Ying Mei, Dandan Wang, Lan Xie, Xiang Xiao, Wei Liu, Willa A. Hsueh, Xian C. Li, Tuo Deng

**Affiliations:** 1 National Clinical Research Center for Endocrine and Metabolic Diseases, Key Laboratory of Diabetes Immunology, Ministry of Education, and Department of Metabolism and Endocrinology, and Metabolic Syndrome Research Center, The Second Xiangya Hospital of Central South University, Changsha, Hunan 410011 China; 2 Department of Pediatrics, Tongji Hospital, Tongji Medical College, Huazhong University of Science and Technology, Wuhan, Hubei 430030 China; 3 Department of Endocrinology, The Second Affiliated Hospital of Guangzhou Medical University, Guangzhou, 510260 China; 4 Henan Key Laboratory of Rare Diseases, Endocrinology and Metabolism Center, The First Affiliated Hospital, and College of Clinical Medicine of Henan University of Science and Technology, Luoyang, 471003 China; 5 Tsinghua University, Intelligent Chinese Medicine Engineering Research Center, Beijing, 100084 China; 6 Immunobiology and Transplant Science Center and Department of Surgery, Houston Methodist Hospital, Houston, TX USA; 7 Department of General Surgery, The Second Xiangya Hospital of Central South University, Changsha, Hunan 410011 China; 8 Diabetes and Metabolism Research Center, Division of Endocrinology, Diabetes and Metabolism, Department of Internal Medicine, The Ohio State University, Columbus, OH 43210 USA; 9 FuRong Laboratory, Changsha, Hunan 410078 China; 10 Clinical Immunology Center, The Second Xiangya Hospital of Central South University, Changsha, Hunan 410011 China

**Keywords:** Metabolic syndrome, Obesity

## Abstract

T cells contribute critically to obesity-induced adipose inflammation and insulin resistance, yet the co-stimulatory signals that govern their activation in adipose tissue remain unclear. Here, we systematically profile co-stimulatory molecules in adipocytes and adipose tissue macrophages and identify OX40 ligand (OX40L) as the most robustly upregulated in obesity. OX40L is also elevated in adipocytes from obese humans. Although macrophage-specific OX40L deletion has no metabolic impact, global OX40 deficiency or adipocyte-specific OX40L deletion reduces Th1 cell accumulation in visceral adipose tissue, attenuates inflammation and improves insulin sensitivity without affecting adiposity. These benefits are reversed by Th1 cell transfer. Therapeutic blockade of OX40L with a neutralizing antibody mimics the protective effects of genetic deletion. Our findings identify adipocyte-derived OX40L as a critical mediator of obesity-associated immune dysfunction and establish it as a targetable checkpoint for tissue-specific immunotherapy in metabolic disease.

## Adipocyte OX40L Drives Inflammation and Insulin Resistance in Obesity

The global rise in obesity presents a significant health challenge, closely linked to chronic low-grade inflammation in visceral adipose tissue (VAT), which contributes to insulin resistance and related diseases. This study identifies the OX40 ligand (OX40L) as a critical co-stimulatory molecule upregulated in adipocytes and macrophages during obesity. Using human and mouse models, the authors demonstrate that adipocyte-derived OX40L is essential for activating Th1 cells in VAT, exacerbating inflammation and insulin resistance. They employed genetic models and OX40L-blocking antibodies to show that inhibiting OX40L reduces VAT inflammation and improves metabolic health. These findings highlight the OX40L-OX40 signalling pathway as a potential therapeutic target for obesity-related metabolic dysfunction. Future research could focus on developing adipocyte-targeted therapies to selectively inhibit OX40L, offering a promising strategy for treating metabolic disorders without compromising systemic immune function.

This summary was initially drafted using artificial intelligence, then revised and fact-checked by the author.

## Introduction

The rising prevalence of obesity poses a significant global challenge^1^, heightening the risk of associated comorbidities such as type 2 diabetes mellitus (T2DM), cardiovascular disease, non-alcoholic steatohepatitis (NASH) and cancer^2^. Chronic low-grade inflammation associated with obesity is a critical mechanistic link between excess adiposity and these serious disorders^3,4^. Among various tissues, visceral adipose tissue (VAT) inflammation plays a pivotal role in the development of insulin resistance and other obesity-related complications, primarily through alterations in immune-cell composition^5–7^. Thus, targeting VAT inflammation represents a promising therapeutic strategy for combating obesity-associated diseases.

A variety of immune cells are involved in obesity-induced VAT inflammation^7,8^. Adipose tissue macrophages (ATMs) are substantially increased in obesity^9^ and serve as key regulators of inflammatory processes^10,11^. However, adipose-resident T cells (ARTs) also play a central role by modulating the phenotypes of pro-inflammatory ATMs and adipocytes. In lean individuals, CD4⁺ T helper 2 (Th2) cells and regulatory T (Treg) cells dominate the ART population and help to maintain an anti-inflammatory microenvironment through the secretion of cytokines such as interleukin-10 (IL-10)^12^. High-fat diet (HFD) feeding induces the expansion of Th1 and CD8^+^ T cells, leading to enhanced interferon gamma (IFNγ) production, which promotes pro-inflammatory macrophage polarization and reduces Th2 and Treg cell abundance^13,14^. Depletion of Th1 or CD8^+^ T cells ameliorates HFD-induced adipose inflammation and insulin resistance^15,16^. Although Th1 and CD8^+^ T cells are now recognized as key components of obesity-induced adipose inflammation, the specific signals that initiate their activation remain elusive. Uncovering these signals would guide new strategies to control obesity-induced inflammation.

T cell activation requires both major histocompatibility complex (MHC)-mediated antigen presentation and co-stimulation. CD4^+^ and CD8^+^ T cells, respectively, recognize antigens presented by MHCII and MHCI, and usually share co-stimulation signals^17^. Our previous work demonstrated that MHCII expression in adipocytes increases during obesity and plays a crucial role in promoting adipose inflammation and insulin resistance^18,19^. HFD-fed *MHCII*^−/−^ mice show reduced Th1 activation and improved insulin sensitivity despite comparable adiposity with wild-type (WT) controls^18,20^. Moreover, adipocyte-specific deletion of MHCII recapitulates these effects, further implicating adipocytes as functional antigen-presenting cells (APCs) in obesity^21^. In parallel, Dr. Lumeng’s group identified ATMs as dominant APCs within the stromal vascular fraction (SVF) of VAT and showed that macrophage-specific MHCII deletion also improved adipose inflammation and insulin sensitivity under HFD feeding^20^. These studies suggest that both adipocytes and ATMs contribute to adipose CD4⁺ T cell activation during obesity through MHCII-dependent antigen presentation. However, antigen recognition alone is insufficient, and co-stimulatory signals are also required for full T cell activation.

Several T cell co-stimulators have been investigated in the context of obesity. CD40, CD80 (B7-1) and CD86 (B7-2) are induced in adipose tissue from obese mice^22,23^. Nevertheless, they are unlikely to co-stimulate ART activation in obesity, because both CD40 and CD80/CD86 show protective effects on HFD-induced adipose inflammation and insulin resistance^23–25^. Both CD40 knockout mice and CD80/CD86 double knockout mice fed a HFD have aggravated inflammation in adipose tissue because of increased CD8^+^ ARTs and ATMs^23,24^ or decreased adipose Tregs^25^, respectively. The 4-1BB ligand is modestly elevated in obese adipose tissue, and although 4-1BB deficiency improves metabolic outcomes, this effect has been attributed to enhanced brown adipose tissue activity and reduced weight gain^26^. The co-stimulatory molecule ICOS is highly expressed on Tregs and limits their VAT accumulation by suppressing CCR3 expression^27^. Although ICOS-deficient mice show improved insulin sensitivity, the regulation of ICOS or ICOSL in adipose tissue during obesity remains unclear^27^. Collectively, these findings underscore the need to identify specific co-stimulatory molecules that directly drive ART activation in obese VAT.

In this study, we identify OX40 ligand (OX40L) as a key co-stimulatory molecule that is markedly upregulated in adipocytes and ATMs during obesity. We demonstrate that adipocyte-derived, but not macrophage-derived, OX40L is essential for Th1 activation in VAT and contributes to obesity-induced insulin resistance. Importantly, treatment with an OX40L-blocking antibody alleviates VAT inflammation and improves metabolic parameters in obese mice. These findings uncover a critical role for adipocyte-T cell OX40L-OX40 signalling in driving obesity-induced immune activation and metabolic dysfunction, and highlight this pathway as a potential therapeutic target.

## Materials and Methods

### Human samples

This study was conducted in compliance with the Declaration of Helsinki and was approved by the Ethics Committee of the Second Xiangya Hospital of Central South University (No. LYF2022207). Written informed consent was obtained from all human donors prior to their enrolment in the study. Human VAT (omental WAT) samples were obtained from two distinct groups: obese donors (BMI ≥ 30 kg/m^2^) eligible for bariatric surgery, and non-obese donors (BMI < 30 kg/m^2^) undergoing non-acute cholecystectomy surgery. Exclusion criteria included fever or infection, renal or neoplastic disease, organ transplantation, steroid use, oestrogen replacement, AIDS, >10% body weight change within the preceding 3 months, or previous diagnosis of type 1 diabetes, haemochromatosis or lipodystrophy. Freshly collected VAT specimens (200 mg tissue blocks) were immediately processed (≤ 2 h post-excision) for mechanical dissociation, followed by collagenase digestion to isolate mature adipocytes. Phenotypic parameters of human donors are listed in Supplementary Table 2.

### Animals

All animal studies were performed in accordance with procedures approved by the Central South University Animal Care and Use Committee. Mice were housed in specific pathogen-free facilities under controlled environmental conditions (22 °C, 50-60% humidity) with a 12-h light/dark cycle. Animals were provided with ad libitum access to water and either a normal diet (ND) (1010001, Jiangsu Xietong Pharmaceutical Bio-engineering Co. Ltd.) or a 60% HFD (D12492, Research Diets, Inc.). 6-week-old C57BL/6 J mice were obtained from Slac Laboratory Animal Inc, whereas 12-week-old C57BL/6 J *ob/ob* (*Lep*^*ob*^*/Lep*^*ob*^) mice were purchased from the Model Animal Research Center of Nanjing University. We acquired *Tnfsf4*^*tm1a*^ embryonic stem cells (clone number: HEPD0717_6_A11) from the European Conditional Mouse Mutagenesis Program (EUCOMM) and generated *Tnfsf4*^*tm1a*^ mice in the Charles River laboratories. We then crossed the *Tnfsf4*^*tm1a*^ mice with FLP transgenic mice (Jackson Laboratory, Stock No.009086) to breed and remove the lacZ-neo cassette, thereby obtaining *Tnfsf4*^*flox/flox*^ mice. *Tnfrsf4* KO mice (Stock No. 012838), *Adipoq*-Cre mice (Stock No.028020), *Lysm*-Cre mice (Stock No.004781) and OT-II (Stock No.004194) mice were obtained from the Jackson Laboratory. AKO mice were generated by breeding *Adipoq*-Cre mice with *Tnfsf4*^*flox/flox*^ mice. Similarly, MKO mice were produced by breeding *Lysm*-Cre mice with *Tnfsf4*^*flox/flox*^ mice. 6-week-old male mice were fed with HFD for 12 weeks to establish the diet-induced obesity (DIO) model. These male mice underwent humane primary euthanasia via carbon dioxide (CO₂) inhalation, followed by cervical dislocation as a secondary confirmatory method, prior to tissue collection.

### Adipose tissue fractionation

Adipose tissues (eWAT and iWAT) were minced and digested with 1 mg/ml type II collagenase (Worthington, Cat#LS004177) and 1% bovine serum albumin at 37 °C for 30 min with gentle shaking at 120 rpm. The mixture was filtered to remove undigested fragments and centrifuged at 300 g for 5 min. The upper layer, containing a mature adipocyte fraction, was aspirated and washed with PBS containing 2 mM EDTA. The pellet (SVF) was washed with PBS and incubated with red blood cell lysis buffer on ice for 5 min. After lysis, the SVF was washed with PBS and collected by centrifugation at 500 g for 5 min. The purified adipocytes and SVF were then prepared for further analysis.

### RNA isolation and quantitative reverse transcription PCR

According to the manufacturer’s instructions, Trizol (Invitrogen, Cat#15596018CN) was used to extract total RNA from cells and tissues. Accurate Biology’s cDNA Synthesis kit (Cat#AG11728) facilitated the reverse transcription of RNA. The SYBR Green Master Mix (Vazyme, Cat#Q511-02) was utilized for Real-Time PCR on the Applied Biosystems ViiA™ 7 Real-Time PCR System. The calculation of normalized mRNA expression in mouse samples utilized β-actin or 36B4 as reference genes, whereas 36B4 was used for human samples. Relative mRNA expression was assessed using the ΔΔCt method. Primer sequences are listed in Supplementary Table 1.

### Flow cytometry

Following standardized adipose tissue fractionation, 60 μl of isolated adipocytes were collected and incubated with anti-CD16/32 antibody (1:100, Biolegend, Cat#101302) for FcR blockade (7 min, room temperature, dark). To identify OX40L^+^ adipocyte population, cells were stained with anti-CD45 (1:200, Biolegend, Cat#103132) and anti-OX40L (1:100, Biolegend, Cat#108810) in PBS containing 1% FBS and 2 mM EDTA (7 min, room temperature, dark). The reaction was quenched with 1 ml PBS, followed by 5 min incubation for phase separation at room temperature. The lower aqueous phase was carefully aspirated using a 1 ml syringe, and the adipocytes were resuspended in 100 μL PBS, transferred to flow cytometry tubes, and stained with 0.6 μL propidium iodide (PI, Biolegend, Cat#421301) for viability assessment prior to flow cytometric analysis. The instrument used for flow cytometry analysis of adipocytes was the NovoCyte Quanteon Flow Cytometer. Before loading, the adipocytes were thoroughly mixed and introduced into the machine at a low flow rate.

The SVF was isolated by centrifugation at 300 g for 5 min at room temperature, separating it from floating adipocytes. For surface-marker detection, cells were first incubated with FcR blocking antibody (1:100, Biolegend, Cat#101302) for 7 min at room temperature, followed by incubation with antibodies against surface markers for 30 min at 4 °C in the dark. After washing with PBS, cell viability was assessed using PI for 5 min at room temperature. For intracellular staining, cells were incubated with Zombie NIR (1:200, Biolegend, Cat#423106) for 7 min at room temperature, and then blocked with anti-CD16/32 antibody (1:100, Biolegend, Cat#101302) and stained with fluorochrome-labelled mAbs against cell-surface antigens for 30 min at 4 °C. Cells were subsequently fixed and permeabilized using the Foxp3/transcription factor staining buffer set (eBioscience, Cat#00-5523-00) according to the manufacturer’s protocol, followed by intracellular staining with fluorochrome-conjugated antibodies for an additional 45 min at 4 °C in the dark. The following mAbs were used: anti-CD45 (1:200, Biolegend, Cat#103132), anti-CD45.1 (1:200, Biolegend, Cat#110728), anti-CD3 (1:200, Biolegend, Cat#100306 and Cat#100236), anti-CD8 (1:200, Biolegend, Cat#100730), anti-CD4 (1:200, Biolegend, Cat#100428), anti-CD11b (1:200, Biolegend, Cat#101222), anti-F4/80 (1:200, Biolegend, Cat#123114), anti-CD11c (1:200, Biolegend, Cat#117308), anti-CD206 (1:200, Biolegend, Cat#141707), anti-Foxp3 (1:200, eBiosicence, Cat#17-5773-82), anti-IFNγ (1:200, Biolegend, Cat#505808; 1:200, BD Bioscience, Cat#566097), anti-IL17A (1:200, Biolegend, Cat#506938), anti-IL4 (1:200, Biolegend, Cat#144806) and anti-Ki67 (1:200, Biolegend, Cat#652410). The absolute number of each CD4⁺ T cell subset per gram of adipose tissue was calculated by multiplying the following parameters: (1) the total number of SVF cells per gram of adipose tissue, (2) the percentage of live cells, (3) the percentage of CD45⁺ leukocytes, (4) the percentage of CD3⁺ T cells, (5) the percentage of CD4⁺ T cells within CD3⁺ lymphocytes and (6) the percentage of the specific CD4⁺ subset (Th1, Th2, Th17 or Treg) within CD4⁺ T cells.

### Immunofluorescence staining

Adipocytes were isolated from mouse epididymal white adipose tissue (eWAT). The cells were washed 3–5 times with PBS containing 2 mM EDTA to remove debris. For staining, 50 μl of adipocyte suspension was transferred to a fresh tube and mixed with 100 μl of PBS. To block Fc receptors, anti-CD16/32 antibody (1:100) was added and incubated at room temperature for 7 min. Subsequently, BODIPY (100 μg/ml, Invitrogen, D3922), Hoechst (10 μg/ml, Beyotime, Cat#C1022) and PE anti-mouse OX40L antibody (1:100, Biolegend, Cat#108806) were added to the mixture and incubated at room temperature for 10 min. After staining, the adipocytes were washed with 1 ml of PBS. The bottom aqueous phase was removed, and the cells were resuspended in 50 μl of PBS. The stained adipocyte suspension was transferred to a confocal dish, and images were acquired using a confocal fluorescence microscope (Zeiss).

### Glucose tolerance test and insulin tolerance test

To conduct glucose tolerance tests (GTTs), mice were fasted for 16 h before receiving an intraperitoneal injection of glucose at a dose of 1 g/kg. At 0, 15, 30, 60, 90 and 120 min, blood samples from the tail vein were measured with a Roche glucometer. For insulin tolerance tests (ITTs), mice were fasted for 6 h and injected with insulin at a dose of 0.75 U/kg. Blood samples taken from the tail vein were measured at 0, 15, 30, 45, 60 and 90 min using a Roche glucometer.

### Fasting insulin measurements

Mice were fasted for 6 hours prior to sample collection. For fasting plasma insulin measurement, blood samples were collected from the retro-orbital sinus into EDTA-coated tubes. Plasma was obtained by centrifugation at 3000 rpm for 15 min, and insulin levels were quantified using a commercial ELISA kit (AiFang biological company, Cat#AF2579-A)) according to the manufacturer’s instructions.

### Western blot

To examine OX40L protein expression, adipocytes, macrophages and SVFs were isolated from eWAT of mice fed either a HFD or ND. To check the insulin signalling, mice were fasted for 6 h prior to intraperitoneal injection with either insulin (4 U/kg) or PBS as a control. Skeletal muscle, liver and eWAT were collected 15 min after injection. Cells and tissues were lysed in RIPA lysis buffer (Beyotime, Cat#P0013B) supplemented with cOmplete Mini Protease Inhibitor Cocktail Tables (Roche, Cat#11836153001) and cOmplete EDTA-free (Sigma-Aldrich, Cat#4693132001). Protein quantification was performed using BCA assays optimized for different sample types: BCA Protein Assay Kit (Beijing Dingguo Changsheng Biotech, Cat# P0398M) for non-adipose tissues and Pierce Microplate BCA Protein Assay kit (Thermo Scientific, Cat#23252) for adipocyte samples, following manufacturers’ protocols. For immunoblotting, 30 μg of total protein were loaded onto 10% SDS-polyacrylamide gels and transferred to PVDF membranes (Merck Millipore, Burlington, MA, USA). After blocking with 5% BSA in PBS for 1 h at room temperature, membranes were incubated overnight at 4 °C with the following primary antibodies against: OX40L (1:1000, AiFang biological, Cat#AF07062), β-actin (1:40000, Sigma-Aldrich, Cat#A5316), p-AKT (1:1000, Cell Signaling Technology, Cat#13038S) and AKT (1:1000, Cell Signaling Technology, Cat#4691S). Following phosphotyrosine detection, membranes were stripped with buffer containing 100 mM β-mercaptoethanol, 2% SDS, and 62.5 mMTris-HCl (pH 7.6) at 50 °C for 30 min, and reincubated with total AKT antibody at 4 °C overnight. Membranes were washed three times (15 min each) with TBST, followed by incubation with anti-HRP secondary antibody (1:20000, Cell Signaling Technology, Cat#7074P2) in 2% BSA for 1 h at room temperature with gentle agitation. The membrane was washed as previously. Clarity Western ECL Substrate (Bio-rad, Cat#1705061) was applied and exposure to film followed. Bio-Rad Image Lab and Image-Pro Plus software were used for analysis.

### Differentiation and adoptive transfer of Th1 cells

Naive CD4^+^ T cells were purified from splenocytes of 6–8-week-old male CD45.1 mice using magnetic bead cell sorting (Miltenyi Biotec, Cat#130-104-453). Cell purity was confirmed by flow cytometry analysis of CD4^+^CD44^low^CD62L^high^ T cell subset. These naïve CD4^+^ T cells were stimulated with 2 μg/ml pre-coated anti-CD3ε antibody (Selleck, Cat#A2104) and 2 μg/ml soluble anti-CD28 antibody (Selleck, Cat#A2108) in culture medium containing 20 ng/ml IL12 (Peprotech, Cat#210-12), 10 ng/ml IL2 (Peprotech, Cat#200-02) and 10 μg/ml anti–IL4 antibody (BD Biosciences, Cat#554432) for 3 days. The culture medium was RPMI 1640 medium (plus 50 mM β-mercaptoethanol) supplemented with 10% FBS, 1% GlutaMax, and 1% Pen/Strep (Gibco, Shanghai, China). The purity of Th1 cells after differentiation was validated by flow cytometry.

Male 8-week-old AKO and AWT mice were maintained on a HFD for 11 weeks prior to receiving 1×10^7^ in vitro-differentiated Th1 cells via intraperitoneal injection. 3 days after the injection, ITTs were performed. 7 days after the injection, the mice were sacrificed. In addition, to verify Th1 cell recruitment, cells from eWAT and spleen were analysed by flow cytometry.

### Adipocyte-naïve CD4^+^ T cell co-culture and antigen-specific T cell activation assays

Adipocytes of AKO and AWT mice fed 12 weeks of HFD were incubated with medium alone or 10 μg/ml OVA peptide (OVA323-339, Sigma, Cat#O1641) at 37 °C on a shaker. Naïve CD4^+^ T cells were purified from the splenocytes of OT-II mice using the MojoSort Mouse CD4 Naïve T Cell Isolation Kit (Miltenyi Biotec, Cat#130-104-453). The pulsed adipocytes were then co-cultured with isolated T cells in RPMI-1640 complete medium supplemented with 10 ng/ml IL2 (Peprotech, Cat#200-02) on a shaker. In some experiments, adipocytes isolated from HFD-fed AWT mice were co-cultured with naïve CD4⁺ T cells from OT-II mice treated with α-OX40L or control IgG2b antibodies. After 3 days, T cells were collected and analysed by flow cytometry, and the Th1 cytokine IFNγ in the culture supernatant was measured by ELISA (R&D Systems, Cat#DY485).

### Adipocyte-differentiated Th1 cell co-culture assay

In vitro differentiated Th1 cells (prepared as described above) were co-cultured with adipocytes isolated from HFD-fed AWT or AKO mice in RPMI-1640 complete medium on a shaker. In some experiments, adipocytes isolated from HFD-fed AWT mice were co-cultured with differentiated Th1 cells treated with α-OX40L or control IgG2b antibodies. After 3 days of co-culture, T cells were harvested for flow cytometric analysis of IFNγ expression, and culture supernatants were collected for ELISA measurement of IFNγ levels.

### Adipocyte–CD8^+^ T cell co-culture assay

CD8⁺ T cells were purified from splenocytes of C57BL/6 J mice using the MojoSort Mouse CD8 T Cell Isolation Kit (BioLegend, Cat#480008) according to the manufacturer’s instructions. To activate CD8⁺ T cells, culture plates were first coated with 2 μg/ml anti-CD3 antibody. Purified CD8⁺ T cells were then cultured in RPMI-1640 complete medium supplemented with 10% FBS, 1% Pen/Strep, 1% GlutaMax, 50 mM β-mercaptoethanol, 2 μg/ml soluble anti-CD28 antibody and 10 ng/ml IL-2. Cells were incubated for 24 h to induce CD8⁺ T cell activation. Activated CD8⁺ T cells were subsequently co-cultured with adipocytes isolated from HFD-fed AWT or AKO mice in complete RPMI-1640 medium on a shaker. After 3 days of co-culture, T cells were harvested and analysed by flow cytometry to determine the frequencies of IFNγ⁺, granzyme B⁺ and perforin⁺ CD8⁺ T cells. Culture supernatants were collected for ELISA measurement of IFNγ levels.

### Bone marrow-derived dendritic cells and naïve CD4^+^ T cell co-culture assay

Bone marrow cells were isolated from the femurs and tibias of C57BL/6 J mice and cultured in RPMI-1640 complete medium supplemented with 10% FBS, 1% Pen/Strep and recombinant mouse GM-CSF (20 ng/ml) to generate bone marrow-derived dendritic cells (BMDCs). Fresh medium containing GM-CSF was added on days 3 and 5. On day 7, non-adherent and loosely adherent cells were collected as immature BMDCs. For antigen presentation assays, BMDCs were incubated with OVA peptide (10 μg/ml) for 2 h at 37 °C to allow antigen loading. Then BMDCs were cocultured with naïve CD4⁺ T cells purified from splenocytes of OT-II mice. Cells were cultured in RPMI-1640 complete medium treated with α-OX40L or control IgG2b antibodies. After 3 days of co-culture, T cells were harvested for flow cytometric analysis of Th1 differentiation by staining intracellular IFNγ. Culture supernatants were collected for ELISA measurement of IFNγ levels.

### Cell proliferation assay

DNA synthesis was quantified using the Click-iT^®^ EdU Flow Cytometry Assay Kits (Invitrogen, Cat#C10424) according to the manufacturer’s protocol. Briefly, mice received intraperitoneal injections of EdU at 20 µg/g. Six hours post-injection, SVF were isolated from mice as described previously. Cells were first stained with appropriate surface markers, washed with PBS, then fixed and permeabilized with 50 µl Component D (provided in the kit) for 15 min at room temperature protected from light. Following additional washes with PBS containing 1% BSA, cells were incubated with 100 μl 1×Click-iT saponin-based permeabilization/wash reagent and 125 μl Click-iT reaction cocktail for 30 min at room temperature in the dark. For intracellular cytokine staining, cells were subsequently treated with 1×permeabilization/wash reagent at 4 °C for 30 min containing anti-IFNγ antibody (1:200, Biolegend, Cat#505808).

### Haematoxylin and eosin staining, Oil Red O staining and immunohistochemistry

Paraffin-embedded liver and skeletal-muscle tissue blocks from mice were sectioned at 5 μm in thickness, deparaffinized in xylene, rehydrated through a graded series of ethanol and stained with haematoxylin and eosin (H&E) according to standard procedures. Images were captured using an Olympus microscope with Olympus imaging software.

For Oil Red O staining, fresh liver and skeletal-muscle tissues were embedded in OCT compound and cryosectioned at a thickness of 8 μm. Sections were fixed in 4% paraformaldehyde for 10 min, rinsed with distilled water, and incubated with freshly prepared Oil Red O working solution for 10–15 min at room temperature. After differentiation in 60% isopropanol and washing with distilled water, nuclei were counterstained with haematoxylin. Images were obtained using an Olympus microscope.

For OX40L immunohistochemistry, paraffin-embedded liver and skeletal muscle sections were heated at 60 °C for 1 h, deparaffinized with xylene and rehydrated through graded alcohols. Endogenous peroxidase activity was blocked with 0.3% H₂O₂ for 20 min. To minimize non-specific staining, sections were blocked with 2% BSA in PBS for 30 min at room temperature and then incubated overnight at 4 °C with primary antibody against OX40L (AiFang biological, Cat#AF07062, 1:200). After washing, sections were incubated with a secondary anti-rabbit antibody (Boster, Cat#BA1054, 1:200) for 1 h at room temperature. Signals were visualized using the DAB peroxidase substrate kit (ZSGB bio, Cat#2L2-9018). Images were captured using an Olympus microscope, and quantitative analysis of staining intensity was performed using Image-Pro Plus 6.0 software.

### ELISA measurement of TNFα and IFNγ

eWAT was isolated from HFD-fed AWT and AKO mice, weighed and cultured in DMEM/F12 medium at 37 °C in a humidified incubator with 5% CO₂ for 24 h. After incubation, culture supernatants were collected and centrifuged to remove debris. The concentrations of TNFα and IFNγ in the supernatants were measured using commercial ELISA kits according to the manufacturers’ instructions. TNFα was quantified using a mouse TNFα ELISA kit (Boster, Cat#EK0527), and IFNγ levels were determined using a mouse IFNγ ELISA kit (R&D Systems, Cat#DY485). Absorbance was measured using a microplate reader, and cytokine concentrations were calculated based on standard curves generated with recombinant cytokine standards.

### OX40L antibody blockade assay

C57BL/6 J mice fed a HFD for 12 weeks were randomly assigned to receive intraperitoneal injections of either an OX40L-blocking monoclonal antibody (BioXcell, Cat#BE0033-1) or an isotype-matched control antibody IgG2b (BioXcell, Cat#BE0090), administered at a dose of 300 μg per mouse, twice weekly. Metabolic assessments, including ITTs and GTTs, were performed at week 16 of HFD feeding. At week 18, mice were euthanized, and T cell subsets in VAT and spleen were profiled by flow cytometry.

### Statistical analysis

All data are expressed as the mean ± SEM. Normal distribution of populations at the 0.05 level was calculated. Unless stated otherwise, significance was assessed using Student’s *t* test for two-group comparisons, whereas ANOVA for multiple-group comparisons. Non-parametric data were evaluated using the Mann–Whitney *U* test. For longitudinal metabolic data (ITT and GTT), two-way repeated-measures ANOVA was performed. All statistical analyses were conducted using SPSS version 25.0, with statistical significance defined as *P* < 0.05. The sample size (*n*) for each experiment is specified in the corresponding figure legend.

## Results

### Obesity induces OX40L expression in adipocytes and adipose tissue macrophages

**Fig. 1:**
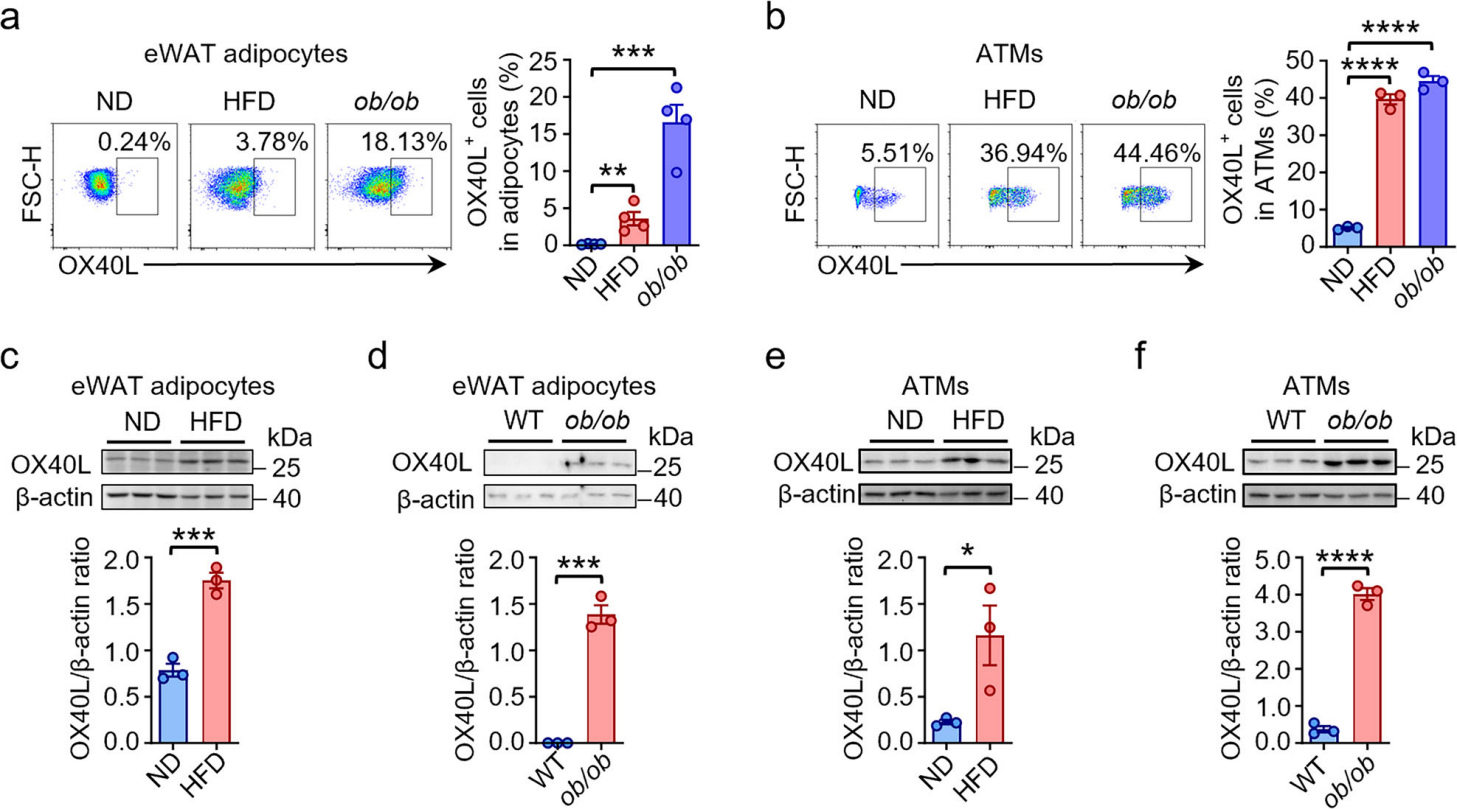
Obesity induces OX40L expression in adipocytes and adipose tissue macrophages. **a** Flow cytometry analysis of OX40L^+^ cell proportions in epididymal white adipose tissue (eWAT) adipocytes from mice on a normal diet (ND), mice on a high-fat diet (HFD) (12 weeks) and *ob/ob* mice (*n* = 4 per group). **b** Flow cytometry analysis of OX40L^+^ cell proportions in eWAT macrophages from mice on a ND, mice on a HFD (12 weeks) and *ob/ob* mice (*n* = 3 per group). **c** Western blot analysis of OX40L protein expression in eWAT adipocytes from mice on a ND and those on a HFD for 12 weeks (*n* = 3 per group). **d** Western blot analysis of OX40L protein expression in eWAT adipocytes from wild type (WT) and *ob/ob* mice (*n* = 3 per group). **e** Western blot analysis of OX40L protein expression in eWAT macrophages from mice on a ND and those on a HFD for 12 weeks (*n* = 3 per group). **f** Western blot analysis of OX40L protein expression in eWAT macrophages from wild type and *ob/ob* mice (*n* = 3 per group). Data represent mean ± SEM. *P < 0.05, **P < 0.01, ***P < 0.001 and ****P < 0.0001. ATMs, adipose tissue macrophages; FSC, forward scatter.

Given that ATMs^20^ and adipocytes^18,19^ function as APCs in obese VAT, we assessed the expression of known T cell co-stimulatory molecules^17^ in these cell types isolated from the epididymal white adipose tissue (eWAT) of lean and diet-induced obesity (DIO) mice. Among the candidates, OX40L was most strongly upregulated in both adipocytes and ATMs from obese mice (Supplementary Fig. 1a, b). Similarly, in transgenic *ob/ob* mice, a genetic model of obesity, OX40L expression was markedly elevated in both adipocytes and ATMs (Supplementary Fig. 1c, d). Notably, HFD feeding specifically enhanced OX40L gene expression in eWAT, without similar upregulation in other examined tissues (Supplementary Fig. 1e), suggesting a VAT-specific regulation in obesity. Flow cytometry analysis revealed a significant increase in OX40L^+^ adipocytes and ATMs in both DIO and *ob/ob* mice (Fig. 1a, b). Given the technical challenges of performing flow cytometry on buoyant and fragile adipocytes, we present the complete gating strategy (Supplementary Fig. 2a), accompanied by bright-field images that depict adipocyte morphology of ND, HFD and *ob/ob* mice (Supplementary Fig. 2b), as well as fluorescence images validating OX40L staining in adipocytes (Supplementary Fig. 2c). Consistently, western blotting confirmed elevated OX40L protein levels in these cells under obese conditions (Fig. 1c–f).

To directly compare OX40L protein abundance in adipocytes and ATMs, we performed immunoblotting of whole eWAT, isolated adipocytes and purified ATMs on the same gel. Under obese conditions, OX40L protein levels in adipocytes were comparable with those detected in ATMs (Supplementary Fig. 1f). Although OX40L mRNA increased more dramatically in ATMs than in adipocytes, mRNA levels do not necessarily correlate linearly with protein abundance owing to post-transcriptional regulation. Moreover, we performed flow cytometry analysis by staining adipocytes and ATMs in the same tube to directly compare OX40L expression. In obese mice, both cell types showed increased OX40L⁺ frequencies (Supplementary Fig. 1g). Notably, although the proportion of OX40L⁺ ATMs increased, their mean fluorescence intensity (MFI) remained largely unchanged (Supplementary Fig. 1h), whereas adipocytes exhibited increases in both the OX40L⁺ frequency and MFI (Supplementary Fig. 1g, h), suggesting elevated per-cell expression in adipocytes.

To examine the translational relevance of these findings, we analysed VAT samples from lean and obese human subjects. Adipocytes from obese individuals exhibited a 2.5- to 4-fold increase in OX40L mRNA levels compared with lean controls (Supplementary Fig. 1i). Owing to limited access to human ATMs, reverse transcriptase-quantitative PCR analysis of these cells was not performed.

To independently validate the cell-type-specific expression of OX40L, we analysed publicly available single-nucleus sequencing (snRNA-seq) datasets^28^. Because snRNA-seq has relatively limited sequencing depth, the detection frequency of OX40L transcripts was low across individual cell types. Nevertheless, comparative analysis between lean and obese samples revealed clear-cell-type-specific patterns of OX40L induction. Among the major antigen-presenting cell populations in adipose tissue, OX40L expression was consistently increased in adipocytes and macrophages in both obese mouse and human visceral adipose tissue, whereas dendritic cells and B cells did not show consistent upregulation across species (Supplementary Fig. 3a–f).

Collectively, these murine experiments, human adipose tissue analyses and independent single-nucleus transcriptomic data demonstrate that OX40L is upregulated in adipocytes and ATMs within obese VAT, implicating the OX40L–OX40 costimulatory axis as a key mediator of obesity-driven ART activation and insulin resistance.

### OX40 deficiency attenuates high-fat-diet-induced adipose inflammation and insulin resistance

**Fig. 2:**
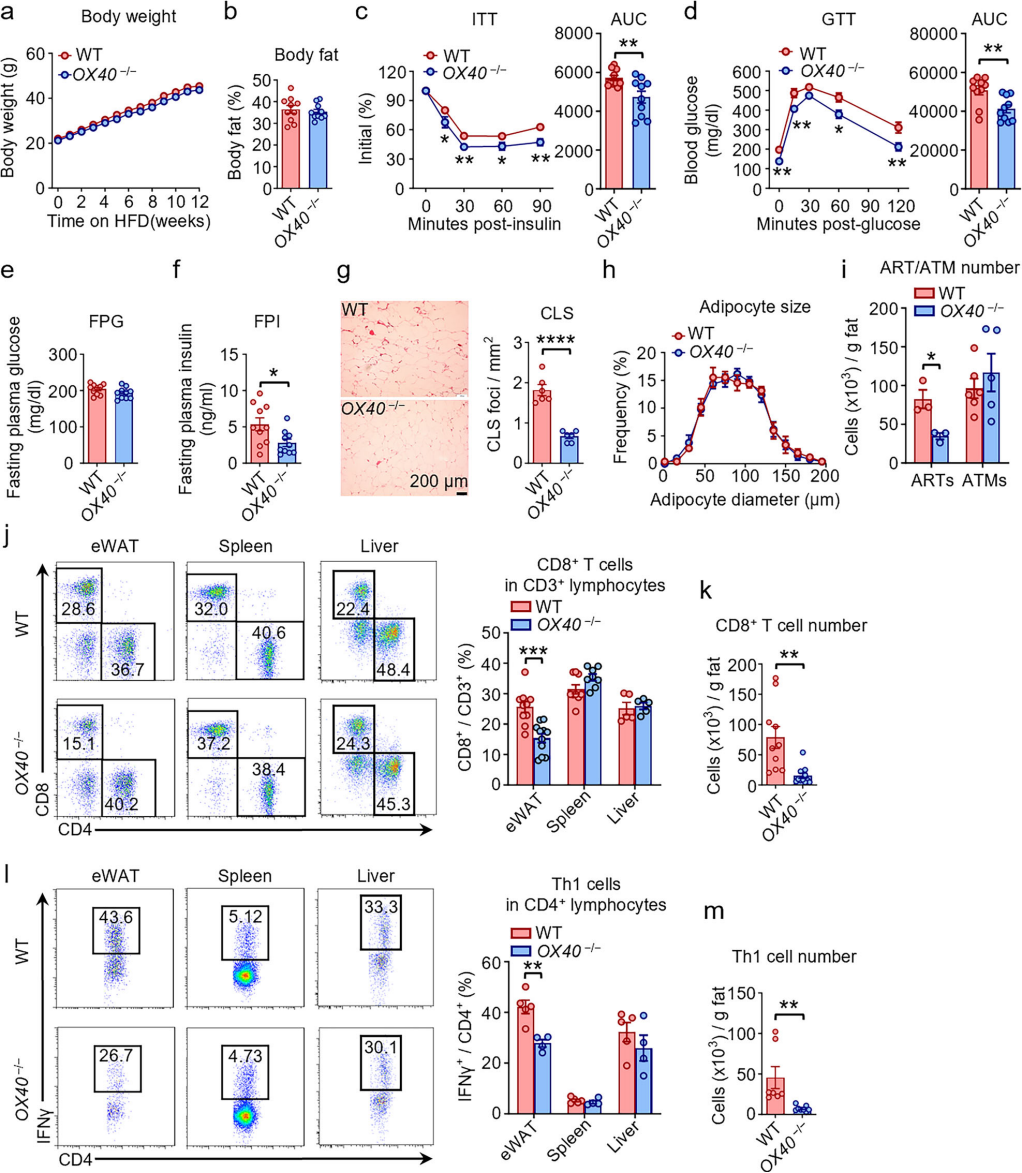
OX40 deficiency attenuates high-fat-diet-induced adipose inflammation and insulin resistance. OX40-deficient (*OX40*^−/−^) and littermate wild type (WT) mice were fed a high-fat diet (HFD) for 12 weeks. **a** Body weight of *OX40*^−/−^ and WT mice (*n* = 10 per group). **b** Body fat of *OX40*^−/−^ and WT mice (*n* = 10 per group). **c** Insulin tolerance test (ITT) results in *OX40*^−/−^ and WT mice, with are under the curve (AUC) quantification shown on the right (*n* = 10 per group). **d** Glucose tolerance test (GTT) results in *OX40*^−/−^ and WT mice, with AUC quantification shown on the right (*n* = 10 per group). **e** Fasting plasma glucose (FPG) levels of *OX40*^−/−^ and WT mice (n = 10 per group). **f** Fasting plasma insulin (FPI) levels of *OX40*^−/−^ and WT mice (*n* = 10 per group). **g** H&E staining of epididymal white adipose tissue (eWAT) from *OX40*^−/−^ and WT mice (scale bar: 200 μm); right panel shows crown-like structure (CLS) density quantification (*n* = 6 per group). **h** Adipocyte diameter quantification in eWAT from *OX40*^−/−^ and WT mice (*n* = 5 per group). **i** Flow cytometry analysis of T cell and macrophage populations in eWAT from *OX40*^−/−^ and WT mice (*n* = 3–6 per group). **j** Flow cytometry analysis of CD8^+^ T cell populations in eWAT, spleen and liver from *OX40*^−/−^ and WT mice (*n* = 5–11 per group). **k** Number of CD8^+^ T cells per gram of eWAT (*n* = 10–11 per group). **l** Flow cytometry analysis of Th1 cell populations in eWAT, spleen and liver from *OX40*^−/−^ and WT mice (*n* = 4–5 per group). **m** Number of Th1 cells per gram of eWAT (*n* = 7–8 per group). Data represent mean ± SEM. *P < 0.05, **P < 0.01 and ***P < 0.001. ART, adipose-resident T cell; ATM, adipose tissue macrophage.

To clarify the role of OX40L–OX40 costimulatory signalling in obesity-induced adipose inflammation and insulin resistance, we examined OX40-deficient (*OX40*^−^^/^^−^) mice under HFD conditions. After 12 weeks of HFD feeding, KO mice and their WT littermates exhibited comparable body weight, fat mass and fasting blood glucose levels (Fig. 2a, b, e). However, *OX40*^−^^/^^−^ mice displayed significantly improved insulin sensitivity, enhanced glucose tolerance and lower fasting insulin levels (Fig. 2c, d, f), indicating that OX40 deficiency mitigates HFD-induced insulin resistance.

Histological analysis revealed a reduced abundance of crown-like structures (CLSs) in the eWAT of obese *OX40*^*−/−*^ mice relative to controls (Fig. 2g). The size of adipocytes was comparable in the two groups (Fig. 2h), suggesting that the reduction in CLS abundance was not attributable to differences in adipocyte hypertrophy-induced cell death. Flow cytometric analysis further revealed a marked reduction in ARTs in the eWAT of *OX40*^*−/−*^ mice, whereas ATM numbers remained unchanged compared with WT controls (Fig. 2i), Flow cytometry gating strategy is shown in Supplementary Fig. 4a and b. Given that T cells are a key component of CLS^12,16,29^, the decline in CLS abundance is likely associated with the reduction in ARTs. Notably, HFD-fed *OX40*^*−/−*^ mice exhibited a significant decrease in the proportion and number of CD8⁺ T and Th1 cells in eWAT, with no corresponding changes observed in the spleen or liver (Fig. 2j–m). To further assess CD4⁺ T cell polarization in adipose tissue, we additionally analysed the frequencies and numbers of Th2 (IL-4⁺), Th17 (IL-17A⁺), and Treg (Foxp3⁺) cells among CD4⁺ T cells in eWAT. OX40 deficiency resulted in a significant reduction in Th17 and Treg cells, whereas Th2 cells remained unchanged (Supplementary Fig. 4c, d). Together, these findings suggest that OX40 signalling broadly supports T cell accumulation or activation within adipose tissue during obesity, thereby promoting adipose inflammation and metabolic dysfunction.

Collectively, these findings demonstrate that OX40 deficiency alleviates adipose tissue inflammation and enhances insulin sensitivity under HFD-induced metabolic stress, highlighting the crucial role of OX40L–OX40 signalling in obesity-associated metabolic dysfunction.

### Macrophage OX40L deficiency does not affect high-fat-diet-induced insulin resistance

To investigate the role of macrophage-derived OX40L in obesity-associated metabolic dysfunction, we generated macrophage-specific OX40L knockout mice (MKO) mice by crossing *Tnfsf4*^*flox/flox*^ mice with *Lysm*-Cre mice. MKO mice were viable and born at expected Mendelian ratios. In HFD-fed MKO mice, OX40L expression was markedly reduced in ATMs and bone-marrow-derived macrophages (BMDMs), but remained unchanged in adipocytes (Supplementary Fig. 5a). Flow cytometry further revealed that the proportion of OX40L^+^ ATMs in MKO mice was significantly lower than in their WT littermates (MWT) (Supplementary Fig. 5b), confirming successful macrophage-specific deletion of OX40L.

Following 12 weeks of HFD feeding, MKO and MWT mice exhibited comparable body weight and adipose tissue mass (Supplementary Fig. 5c–f). There were no significant differences in insulin levels (Supplementary Fig. 5g–j), indicating that macrophage-specific OX40L deficiency does not affect HFD-induced insulin resistance.

To determine how macrophage-derived OX40L affects T cell responses in adipose tissue, we analysed T cell subsets in eWAT and spleen from MKO mice and their control littermates. Flow cytometry showed that the proportions of CD4⁺ and CD8⁺ T cells within CD3⁺ lymphocytes were comparable in MWT and MKO mice in both eWAT and spleen (Supplementary Fig. 6a). Likewise, the frequencies of Th1, Th2 and Th17 cells were not significantly altered (Supplementary Fig. 6b–d). In contrast, Treg cells were significantly reduced in the eWAT of MKO mice, whereas Treg abundance in the spleen remained unchanged (Supplementary Fig. 6e).

Interestingly, despite the reduction of Tregs in adipose tissue, MKO mice did not display detectable metabolic changes. This may reflect the fact that Treg abundance in obese adipose tissue is already reduced compared with lean conditions^12^, such that the additional decrease in MKO mice is insufficient to further aggravate metabolic dysfunction. Together, these findings suggest that macrophage-derived OX40L may preferentially influence Treg homeostasis in adipose tissue.

### Adipocyte OX40L deficiency attenuates high-fat-diet-induced adipose inflammation and insulin resistance

To determine the role of adipocyte-derived OX40L in obesity-induced adipose inflammation and insulin resistance, we generated adipocyte-specific OX40L knockout mice (AKO) by crossing *Tnfsf4*^*flox/flox*^ mice with *Adipoq*-Cre mice. The AKO mice were viable, born at expected Mendelian ratios, and exhibited no discernible morphological differences from their AWT (*Tnfsf4*^*flox/flox*^) littermates. As anticipated, OX40L expression was reduced in all examined adipose depots of HFD-fed AKO mice compared with AWT controls, with no changes detected in non-adipose tissues (Supplementary Fig. 7a). OX40L reduction was specific to adipocytes, with no changes in the SVF or BMDMs (Supplementary Fig. 7b). The protein levels of OX40L in adipocytes were consistently and substantially lower in AKO mice than in AWT mice (Supplementary Fig. 7c, d), whereas OX40L levels in SVF remained unchanged in the groups (Supplementary Fig. 7c). These results confirm the successful generation of a mouse model with adipocyte-specific OX40L deletion.

AKO and AWT littermates were fed either ND or HFD for 12 weeks and analysed for differences in metabolic phenotypes. Under ND conditions, AKO mice exhibited similar body weight, insulin sensitivity and glucose tolerance to AWT controls (Supplementary Fig. 8a–e). Additionally, the frequency of CD4^+^ T, CD8^+^ T, Th1, Th2, Th17 and Treg cells remained unchanged in both eWAT and the spleen of the AKO group compared with the AWT group (Supplementary Fig. 8f–j).

**Fig. 3:**
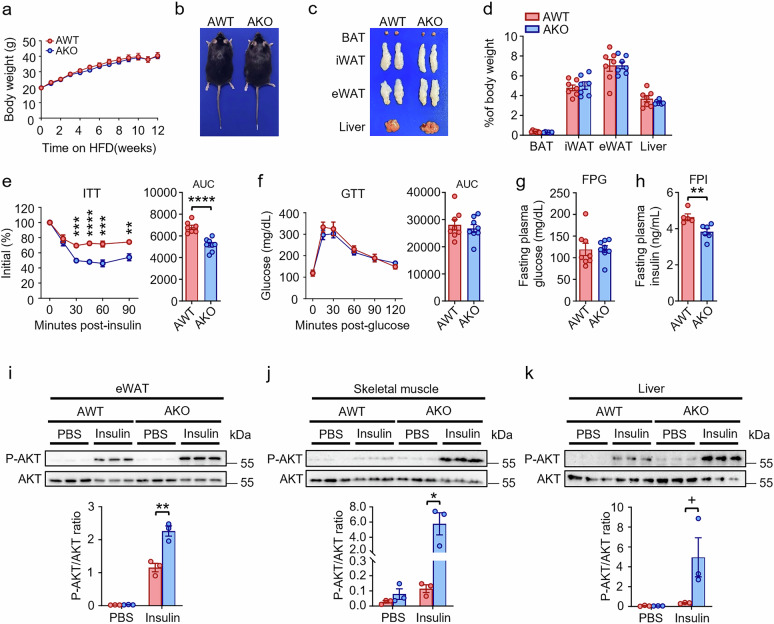
Adipocyte-specific OX40L deficiency attenuates high-fat-diet-induced insulin resistance. Adipocyte-specific OX40L knockout (AKO) and littermate control (AWT) mice were fed a high-fat diet (HFD) for 12 weeks. **a** Body weight of AKO and AWT mice (*n* = 7–8 per group). **b** Representative images of AKO and AWT mice. **c** Representative images of brown adipose tissue (BAT), inguinal white adipose tissue (iWAT), epididymal white adipose tissue (eWAT) and liver from AKO and AWT mice. **d** Weight percentages of BAT, iWAT, eWAT and liver relative to body weight (*n* = 6–7 per group). **e** Insulin tolerance test (ITT) results for AKO and AWT mice (*n* = 7–8 per group). **f** Glucose tolerance test (GTT) results for AKO and AWT mice (*n* = 8 per group). **g** FPG levels of AKO and AWT mice (*n* = 8 per group). **h** FPI levels of AKO and AWT mice (*n* = 5–6 per group). Western blot analysis and quantification of AKT and p-AKT protein levels in eWAT (**i**), skeletal muscle (**j**) and liver (**k**) from AKO and AWT mice after i.p. injection of PBS or insulin (4 U/kg body weight) for 15 min (*n* = 3 per group). Data represent mean ± SEM. *P < 0.05, **P < 0.01 and ****P < 0.0001.

Under HFD conditions, AKO mice showed comparable adiposity and glucose tolerance to AWT controls (Fig. 3a–d, f–g), but exhibited lower fasting insulin levels and improved insulin sensitivity (Fig. 3e, h). Western blotting of eWAT, skeletal muscle and liver demonstrated enhanced post-insulin AKT phosphorylation in AKO mice (Fig. 3i–k), indicating improved insulin responsiveness. Previous studies have shown that adipose tissue inflammation promotes ectopic lipid accumulation in peripheral metabolic organs such as the liver and skeletal muscle, thereby contributing to insulin resistance^30^. To explore whether improved insulin sensitivity in peripheral tissues could be linked to altered lipid metabolism, we examined lipid accumulation in liver and skeletal muscle of HFD-fed AWT and AKO mice. H&E staining and Oil Red O staining revealed markedly reduced lipid accumulation in both liver and skeletal muscle in AKO mice (Supplementary Fig. 9a). Consistent with this observation, expression of genes involved in fatty acid synthesis, fatty acid uptake and lipid droplet formation was significantly reduced in skeletal muscle and liver (Supplementary Fig. 9b, c). Importantly, immunohistochemical analysis showed no difference in OX40L expression in liver or skeletal muscle between AWT and AKO mice (Supplementary Fig. 9d, e), suggesting that the metabolic improvements in these tissues may be secondary to reduced adipose inflammation rather than direct OX40L signalling.

**Fig. 4:**
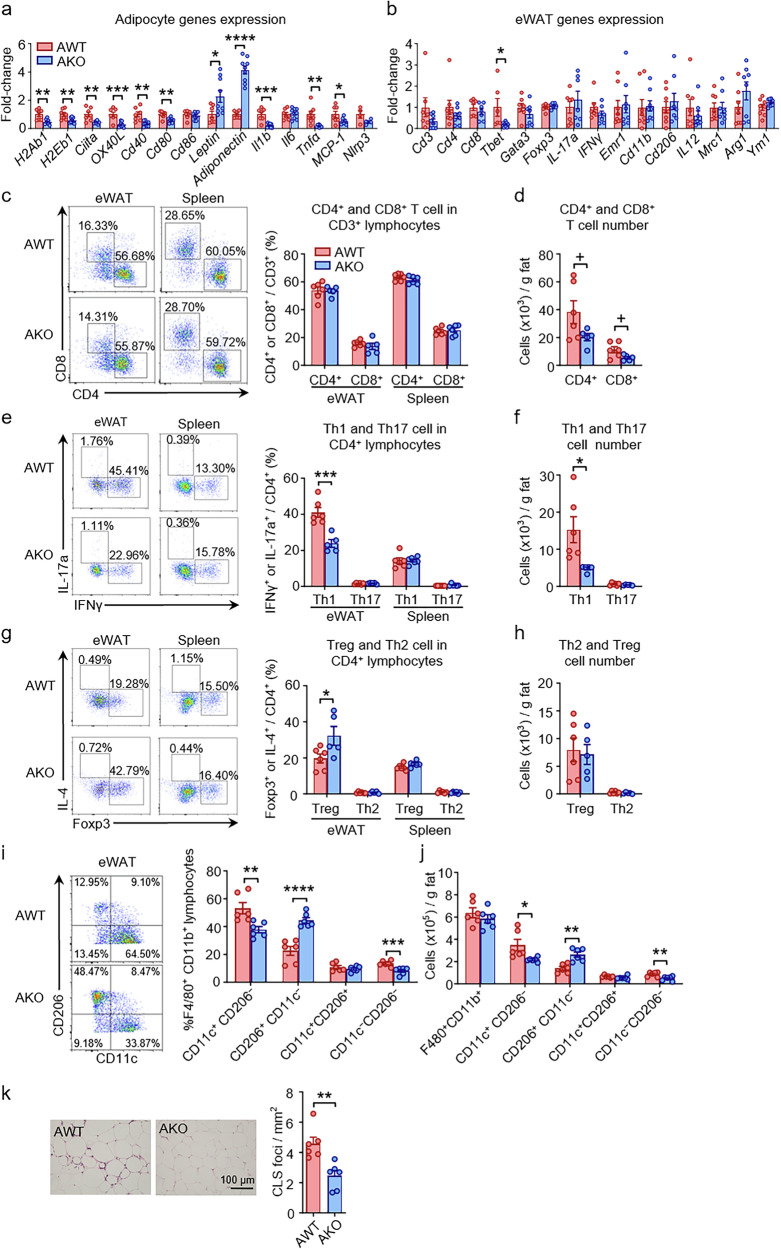
Adipocyte-specific OX40L deficiency attenuates adipose inflammation in obese mice. Adipocyte-specific OX40L knockout (AKO) and littermate control (AWT) mice were fed a high-fat diet (HFD) for 12 weeks. **a** Reverse transcriptase quantitative (RT-qPCR) analysis of gene expression in epididymal white adipose tissue (eWAT) adipocytes from AKO and AWT mice (*n* = 4–9 per group). **b** RT-qPCR analysis of gene expression in eWAT from AKO and AWT mice (*n* = 6–8 per group). **c** Flow cytometry analysis of CD4^+^ and CD8^+^ T cell populations in eWAT and spleen from AKO and AWT mice (*n* = 5–6 per group). **d** Number of CD4^+^ and CD8^+^ T cells per gram of eWAT (*n* = 5–6 per group). **e** Flow cytometry analysis of Th1 and Th17 cell populations in eWAT and spleen from AKO and AWT mice (*n* = 5–6 per group). **f** Number of Th1 and Th17 cells per gram of eWAT (*n* = 5–6 per group). **g** Flow cytometry analysis of Treg and Th2 cell populations in eWAT and spleen from AKO and AWT mice (*n* = 5–6 per group). **h** Number of Treg and Th2 cells per gram of eWAT (*n* = 5–6 per group). **i** Flow cytometry analysis of macrophage subset populations in eWAT from AKO and AWT mice (*n* = 6 per group). **j** Number of total macrophages (F4/80^+^CD11b^+^) and macrophage subsets per gram of eWAT (*n* = 6 per group). **k** H&E staining of eWAT from AKO and AWT mice (scale bar: 100 μm); right panel shows CLS density quantification (*n* = 6 per group). Data represent mean ± SEM. ^+^*P* < 0.1, *P < 0.05, **P < 0.01 and ***P < 0.001. CLS, crown-like structure.

Consistent with their improved insulin sensitivity, AKO mice exhibited reduced expression of pro-inflammatory genes and increased expression of anti-inflammatory genes in adipocytes (Fig. 4a). Specifically, they showed reduced expression of MHCII-related genes (*H2Ab1*, *H2Eb1* and *Ciita*), T cell co-stimulatory genes (*OX40L*, *Cd40* and *Cd80*) and inflammatory factors (*Il1b*, *Tnfα* and *MCP-1*), along with increased expression of the adipokine genes (*leptin* and *adiponectin*). Our previous work showed that IFNγ induces MHCII-related gene expression in adipocytes^18^. Consistently, IFNγ levels in eWAT culture supernatants were significantly reduced in AKO mice (Supplementary Fig. 7e), providing a mechanistic explanation for the decreased expression of MHCII-related genes. In addition, because TNFα suppresses leptin transcription in adipocytes^31^, we measured TNFα levels in eWAT culture supernatants and found them significantly reduced in AKO mice (Supplementary Fig. 7f), which likely explains the increased leptin mRNA expression despite unchanged adipose mass and adipocyte size. Additionally, the Th1 transcription factor *Tbet* was significantly lower in the eWAT of AKO mice than in that of AWT mice, whereas macrophage-related gene expression remained unchanged in the two groups (Fig. 4b).

Flow cytometry analysis of eWAT showed that adipocyte-specific OX40L deletion did not significantly alter the proportion of CD8⁺ T cells and caused only a modest reduction in their number (Fig. 4c, d). Moreover, the expression of IFNγ and FasL in adipose CD8⁺ T cells was comparable in HFD-fed AWT and AKO mice (Supplementary Fig. 10a–d). Consistently, co-culture of adipocytes isolated from HFD-fed AWT or AKO mice with purified CD8⁺ T cells revealed no differences in the frequencies of IFNγ⁺, granzyme B⁺, or perforin⁺ CD8⁺ T cells (Supplementary Fig. 10e–h), indicating that adipocyte-derived OX40L does not directly regulate CD8⁺ T cell effector function. In contrast, although the proportion of CD4⁺ T cells remained unchanged in AKO mice, their absolute number was reduced in eWAT (Fig. 4c, d). Further analysis of CD4⁺ T cell subsets revealed that Th1 cells were significantly reduced in eWAT but not in the spleen (Fig. 4e, f). Conversely, Treg frequency increased in eWAT, although total Treg numbers remained unchanged (Fig. 4g, h), whereas Th2 and Th17 cells were unaffected (Fig. 4e–h). Furthermore, although the total macrophage abundance in eWAT was comparable in AWT and AKO mice (Fig. 4j), further analysis revealed a shift in macrophage polarization. Specifically, both the proportion and absolute number of M1-like macrophages were reduced in AKO mice, whereas M2-like macrophages were increased (Fig. 4i, j). And fewer CLSs were observed in eWAT of AKO mice (Fig. 4k). Together, these findings indicate that adipocyte-specific OX40L deletion mitigates HFD-induced adipose inflammation and insulin resistance.

### Adipocyte-derived OX40L promotes Th1 accumulation and drives insulin resistance

**Fig. 5:**
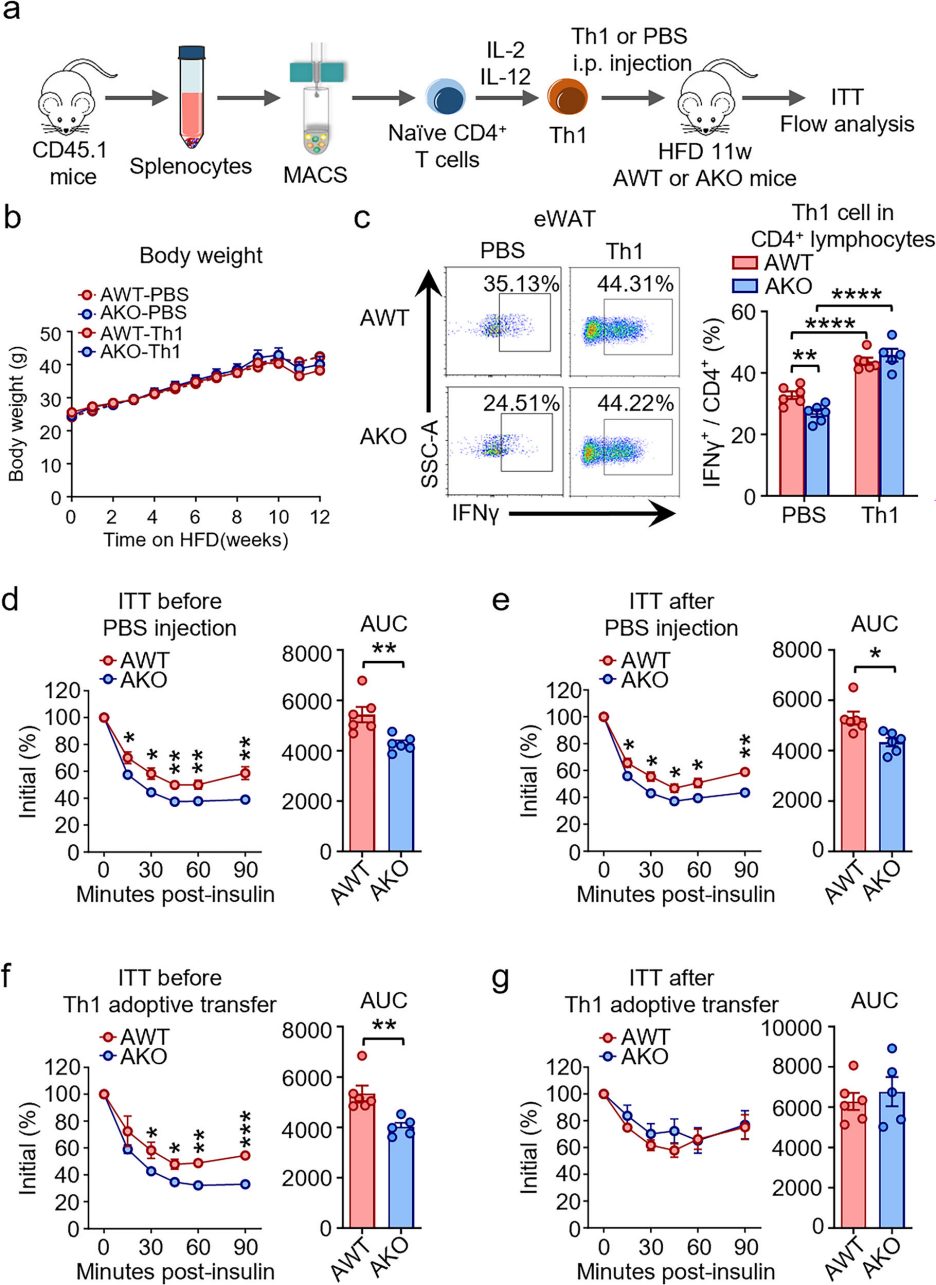
Adoptive transfer of Th1 cells eliminates metabolic benefits in adipocyte-specific OX40L knockout mice under a high-fat diet. **a** Schematic of Th1 cell adoptive transfer experiment. Adipocyte-specific OX40L knockout (AKO) and littermate control (AWT) mice were fed a high-fat diet (HFD) for 12 weeks; Th1 cells or PBS were injected i.p. at HFD 11 weeks, followed by insulin tolerance test (ITT) at day 3 and sacrifice at day 7. **b** Body weight of AKO and AWT mice (*n* = 5–6 per group). **c** Flow cytometry analysis of Th1 cell populations in epididymal white adipose tissue (eWAT) from AKO and AWT mice after PBS or Th1 cell injection (*n* = 5–6 per group). **d** ITT results before PBS injection (*n* = 6 per group). **e** ITT results after PBS injection (*n* = 6 per group). **f** ITT results before Th1 cell transfer (*n* = 5–6 per group). **g** ITT results after Th1 cell transfer (*n* = 5–6 per group). Data represent mean ± SEM. *P < 0.05, **P < 0.01, ***P < 0.001 and ****P < 0.0001. AUC, area under the curve; MACS, magnetic-activated cell sorting.

To determine whether the reduction in adipose Th1 cells observed in HFD-fed AKO mice was responsible for their improved insulin resistance, obese AWT and AKO mice were intraperitoneally injected with inflammatory Th1 cells or PBS, and their insulin responsiveness was assessed via insulin tolerance test (ITT) (Fig. 5a). No significant differences in weight gain were observed between AWT and AKO mice before or after PBS or Th1 cells transfer (Fig. 5b). To track transferred cells, CD45.1⁺ donor Th1 cells were quantified in eWAT and spleen. Flow cytometry revealed that CD45.1⁺ cells were markedly enriched in eWAT compared with the spleen (Supplementary Fig. 11a), indicating preferential accumulation in adipose tissue. Moreover, after transferring equal numbers of fully differentiated CD45.1⁺ Th1 cells, a larger fraction of the transferred Th1 cells accumulated in AKO eWAT (Supplementary Fig. 11b), likely due to increased niche availability. Following adoptive transfer, AKO mice exhibited a marked increase in the proportion of Th1 cells within eWAT, restoring their levels to those observed in AWT mice after Th1 cells transfer (Fig. 5c), thus confirming successful Th1 cell reconstitution. Whether before or after the injection of PBS or before the transfer of Th1 cells, AKO mice displayed significantly enhanced insulin sensitivity compared with AWT mice (Fig. 5d–f). However, following Th1 cell transfer insulin sensitivity was indistinguishable between AWT and AKO mice, indicating that restoration of Th1 cells reversed the metabolic improvement in AKO mice (Fig. 5g).

**Fig. 6:**
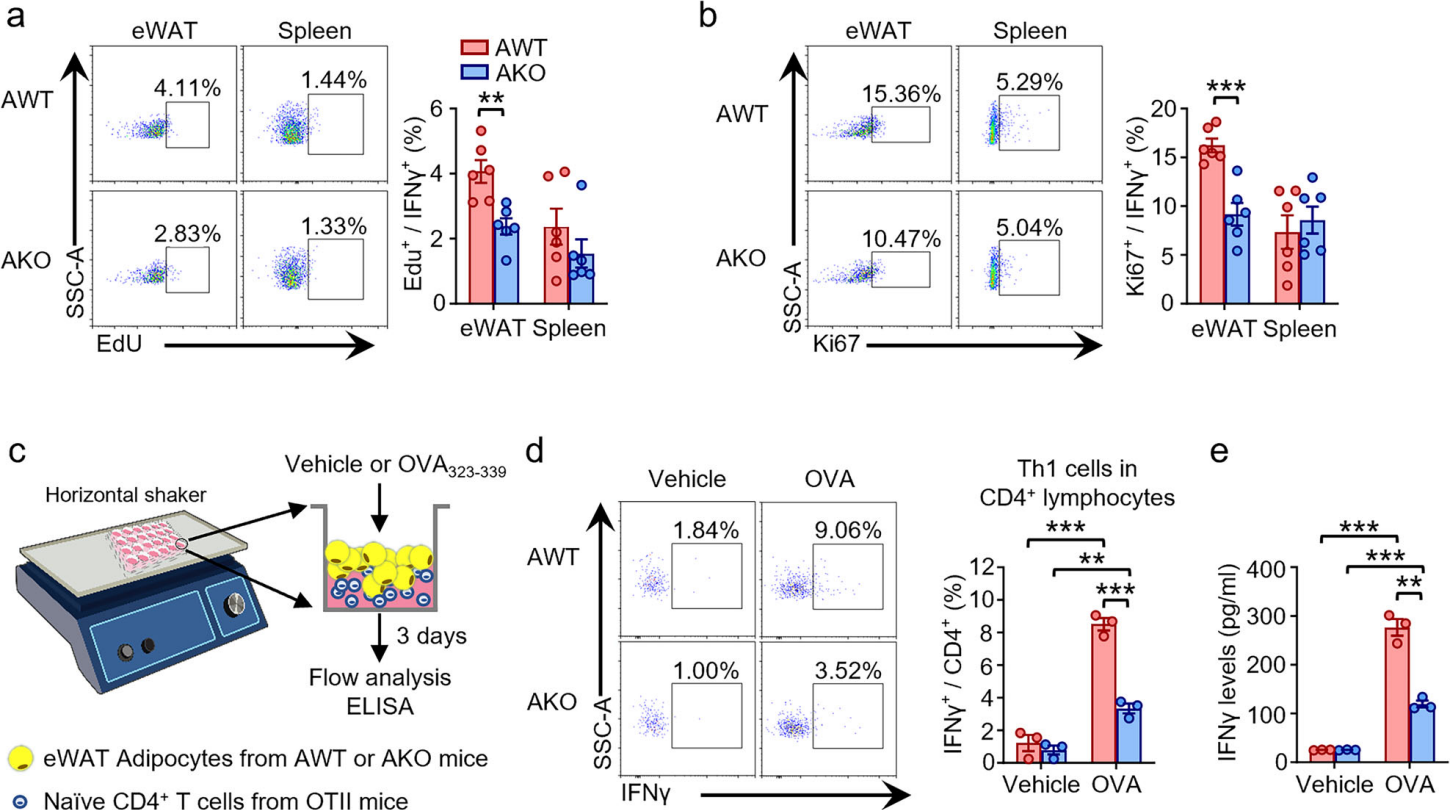
Adipocyte OX40L–OX40 signalling drives Th1 cell differentiation in adipose tissue. **a** Flow cytometry analysis of EdU^+^ Th1 cell populations in epididymal white adipose tissue (eWAT) and spleen from 12-week high-fat-diet (HFD)-fed adipocyte-specific OX40L knockout (AKO) and littermate control (AWT) mice (*n* = 6 per group). **b** Flow cytometry analysis of Ki67^+^ Th1 cell populations in eWAT and spleen from 12-week HFD-fed AKO and AWT mice (*n* = 6 per group). **c** Schematic of adipocyte–naïve CD4^+^ T cell co-culture experiment. **d** Flow cytometry analysis of Th1 cell proportions in co-culture systems (*n* = 3 per group). **e** ELISA quantification of IFNγ levels in co-culture supernatants (*n* = 3 per group). Data represent mean ± SEM. **P < 0.01 and ***P < 0.001.

To further investigate the mechanism underlying the reduction of adipose Th1 cells in AKO mice, we examined the proliferation, differentiation and activation of these cells. For proliferation analysis, we used two markers, 5-ethynyl-2’-deoxyuridine (EdU) incorporation and Ki67 staining. Flow cytometry revealed that the proportions of EdU^+^ and Ki67^+^ Th1 cells in eWAT were significantly lower in AKO mice than in AWT mice (Fig. 6a, b), indicating that reduced proliferation contributes to the decline of Th1 cells in eWAT. Next, to assess adipocyte-mediated Th1 differentiation, we co-cultured CD45-depleted adipocytes from AKO and AWT mice with naïve CD4^+^ T cells from OT-II mice, with or without OVA (Fig. 6c). After 3 days, IFNγ levels in the culture supernatant were measured by ELISA, and Th1 differentiation was analysed by flow cytometry. In the absence of OVA, both AKO and AWT adipocytes exhibited minimal Th1 cell differentiation. However, in the presence of OVA, AKO adipocytes showed a significantly reduced capacity to promote Th1 differentiation (Fig. 6d), which was associated with a marked reduction in IFNγ production (Fig. 6e). Consistently, co-culture of adipocytes from HFD-fed AWT mice with naïve CD4⁺ T cells from OT-II mice in the presence of OX40L blocking antibody (α-OX40L) markedly reduced Th1 differentiation and IFNγ production (Supplementary Fig. 12a, b), further supporting an important role for adipocyte OX40L in Th1 differentiation. However, co-culture of fully differentiated Th1 cells with adipocytes from HFD-fed AWT or AKO mice produced no differences in Th1 frequency, IFNγ expression (MFI) or IFNγ secretion (Supplementary Fig. 12c–e). Similarly, when in vitro-differentiated Th1 cells were co-cultured with adipocytes in the presence of α-OX40L antibody, neither the proportion of IFNγ⁺ cells nor IFNγ secretion was affected (Supplementary Fig. 12f–h). These findings indicate that adipocyte-derived OX40L primarily regulates Th1 differentiation and accumulation rather than the effector function of mature Th1 cells. In summary, these results show that the reduction of Th1 cells in AKO eWAT results from both impaired proliferation and diminished differentiation.

### OX40L-blocking antibody reshapes adipose CD4^+^ T cell responses and improves insulin sensitivity

**Fig. 7:**
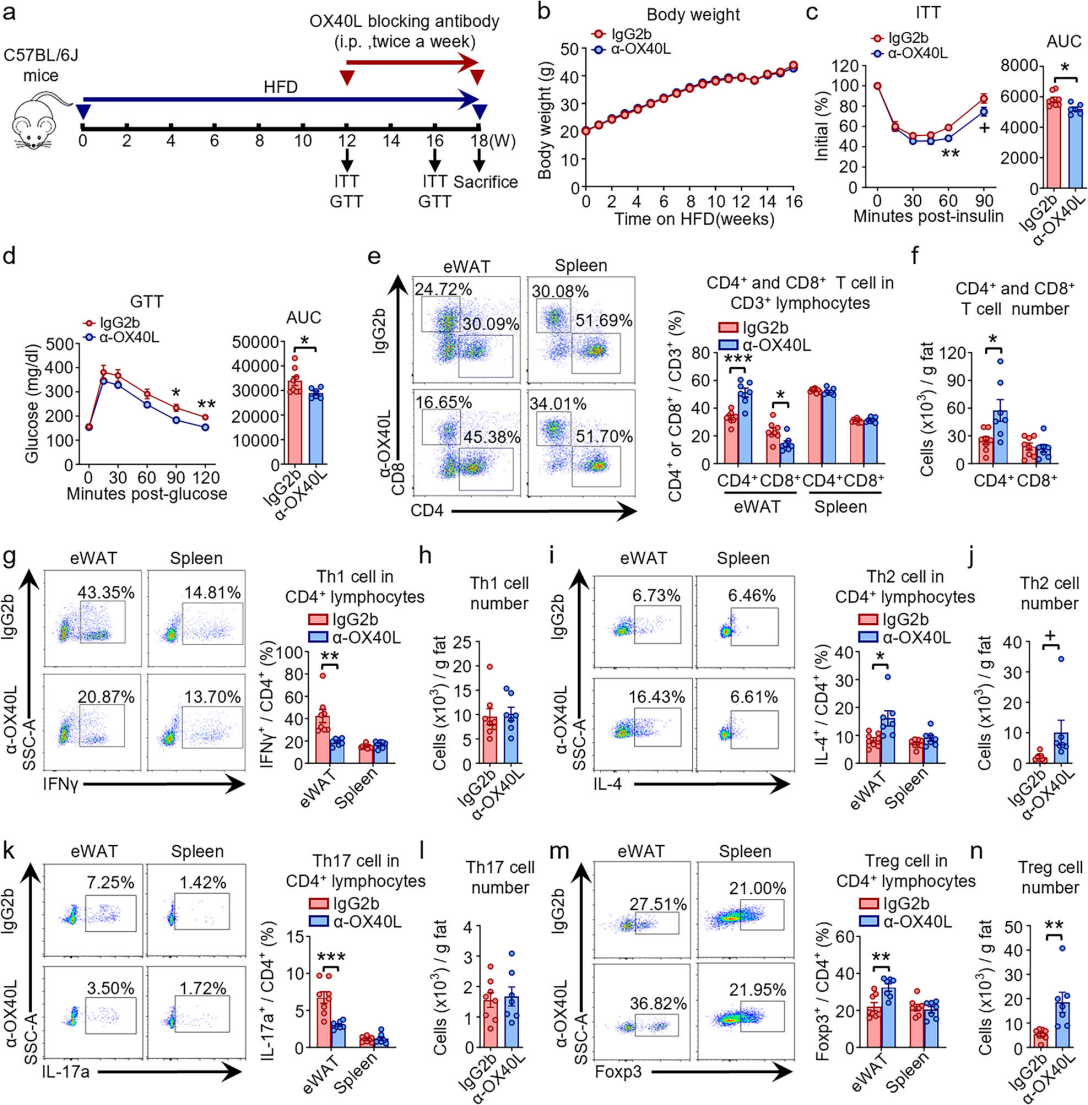
OX40L blocking antibody reshapes adipose CD4^+^ T cell responses and improves insulin sensitivity. **a** Schematic of OX40L-blocking antibody treatment. **b** Body weight of α-OX40L and IgG2b-treated mice (*n* = 7–8 per group). **c** Insulin tolerance test (ITT) results after α-OX40L or IgG2b treatment for 4 weeks (*n* = 6–8 per group). **d** Glucose tolerance test (GTT) results after α-OX40L or IgG2b treatment for 4 weeks (*n* = 6–8 per group). **e** Flow cytometry analysis of CD4^+^ and CD8^+^ T cell populations in epididymal white adipose tissue (eWAT) and spleen after α-OX40L or IgG2b treatment for 6 weeks (*n* = 7–8 per group). **f** Number of CD4^+^ and CD8^+^ T cells per gram of eWAT (*n* = 7–8 per group). **g** Flow cytometry analysis of Th1 cell populations in eWAT and spleen after α-OX40L or IgG2b treatment for 6 weeks (*n* = 7–8 per group). **h** Number of Th1 cells per gram of eWAT (*n* = 7–8 per group). **i** Flow cytometry analysis of Th2 cell populations in eWAT and spleen after α-OX40L or IgG2b treatment for 6 weeks (*n* = 7–8 per group). **j** Number of Th2 cells per gram of eWAT (*n* = 7–8 per group). **k** Flow cytometry analysis of Th17 cell populations in eWAT and spleen after α-OX40L or IgG2b treatment 6 weeks (*n* = 7–8 per group). **l** Number of Th17 cells per gram of eWAT (*n* = 7–8 per group). **m** Flow cytometry analysis of Treg cell populations in eWAT and spleen after α-OX40L or IgG2b treatment for 6 weeks (*n* = 7–8 per group). **n** Number of Treg cells per gram of eWAT (*n* = 7–8 per group). Data represent mean ± SEM. ^+^*P* < 0.1, *P < 0.05, **P < 0.01 and ***P < 0.001. AUC, area under the curve; HFD, high-fat diet.

To evaluate the therapeutic potential of OX40L inhibition, we treated HFD-induced obese mice with either an OX40L blocking antibody (α-OX40L) or an isotype control antibody (IgG2b) (Fig. 7a). Body weight gain and composition did not differ significantly between α-OX40L- and IgG2b-treated mice during HFD feeding (Fig. 7b, Supplementary Fig. 13a, b). Similarly, glucose and insulin tolerance were comparable in the two groups before treatment (Supplementary Fig. 13c, d). However, following treatment, α-OX40L mice exhibited significantly improved insulin sensitivity and glucose tolerance compared with IgG2b controls (Fig. 7c, d). Fasting insulin levels were markedly reduced in α-OX40L mice, whereas fasting glucose levels remained unchanged (Supplementary Fig. 13e, f).

In eWAT, α-OX40L treatment increased the proportion of CD4^+^ T cells while decreasing the proportion of CD8^+^ T cells compared with IgG2b treatment (Fig. 7e), a phenomenon not observed in the spleen. The absolute number of CD4^+^ T cells in eWAT was significantly higher in α-OX40L-treated mice than in IgG2b-treated mice, whereas no significant difference was detected in absolute CD8^+^ T counts (Fig. 7f). Additionally, α-OX40L treatment significantly reduced the proportion of Th1 and Th17 cells in eWAT compared with IgG2b treatment, whereas their absolute numbers remained largely unchanged (Fig. 7g, h, k, l). In contrast, both the proportion and number of Th2 and Treg cells were significantly increased in adipose tissue following OX40L blockade (Fig. 7i, j, m, n). These changes were largely restricted to eWAT and were not observed in the spleen. These changes indicate a shift in the adipose CD4⁺ T cell landscape towards a less inflammatory immune profile, characterized by reduced Th1/Th17 dominance and increased Th2 and Treg responses. Given the established roles of Th2 and Treg cells in suppressing adipose inflammation and promoting metabolic homeostasis, the expansion of these populations probably contributes to the improved GTT and ITT observed after α-OX40L treatment. Importantly, systemic OX40L blockade differs mechanistically from adipocyte-specific OX40L deletion. The α-OX40L antibody acts broadly on multiple OX40L-expressing cell types and immune compartments, thereby inducing global immune modulation. In contrast, adipocyte-specific OX40L deletion selectively disrupts adipocyte–T cell interactions in adipose tissue, leading to a marked reduction in Th1 accumulation without major increases in Th2 or Treg populations.

Taken together, these findings indicate that systemic OX40L blockade improves metabolic outcomes through broader immune remodelling, whereas adipocyte-derived OX40L specifically drives Th1-mediated adipose inflammation and insulin resistance.

## Discussion

Th1 cells play a pivotal role in driving obesity-induced adipose tissue inflammation and insulin resistance^15,32,33^. Understanding the mechanisms underlying Th1 cell accumulation in adipose tissue during obesity is critical for developing therapies against obesity-associated metabolic disorders. The pathogenic expansion of Th1 cells in VAT during obesity can result from multiple mechanisms. For instance, leptin is a key initiator of the adipose inflammatory cascade, with its receptor expression significantly upregulated on ARTs in the obese state^34^. Hyperleptinaemia, driven by adipose tissue expansion, promotes IFNγ secretion by T cells, skewing differentiation towards Th1 while suppressing Th2 responses^35,36^. Our previous work demonstrated that leptin-stimulated IFNγ production in ARTs induces adipocyte MHCII expression, enabling adipocytes to act as APCs that further amplify Th1 activation^18^. These findings establish that obese adipocytes provide MHCII-mediated antigen presentation to promote Th1 activation. More recently, we identified that adipose stem cells (ASCs) orchestrate T cell infiltration into VAT during the early phase of HFD-induced obesity through TNFα/NF-κB-dependent secretion of CCL5, a potent chemokine that attracts T cells^37^. However, targeting these mechanisms lacks specificity for Th1 suppression and may inadvertently disrupt systemic immune homeostasis. Therefore, more selective molecular strategies that specifically inhibit Th1 cells within obese adipose tissue are urgently needed.

Accumulating preclinical and clinical evidence identifies co-stimulatory molecules as promising therapeutic targets owing to their spatiotemporally regulated expression^38–40^. Unlike broadly active immune mediators such as MHC molecules, co-stimulatory signals are selectively upregulated following T cell receptor engagement, allowing targeted immunomodulation with reduced off-target effects^41,42^. These molecules act as critical secondary signals that determine the magnitude and polarization of immune responses, offering unique flexibility to modulate immunity as needed.

In this study, we systematically examined the expression profiles of all known T cell co-stimulators in adipocytes and ATMs and found that OX40L is the most prominently upregulated in both cell types under obese conditions. Genetic ablation of OX40 signalling, either through global OX40-knockout (*OX40*^*−/−*^) (Fig. 2) or adipocyte-specific OX40L deletion (OX40L-AKO) (Fig. 3 and Fig. 4), conferred substantial protection against obesity-induced adipose inflammation and systemic insulin resistance. This phenotype was therapeutically recapitulated by treatment with an OX40L-neutralizing antibody, which alleviated obesity-associated metabolic dysfunction (Fig. 7). These results indicate that adipocytes provide essential co-stimulatory signals through the OX40L–OX40 axis to sustain Th1 differentiation and accumulation in VAT and drive adipose inflammation in obesity. Notably, although OX40L expression was strongly induced in macrophages (Fig. 1 and Supplementary Fig. 1), macrophage-specific OX40L knockout (OX40L-MKO) mice did not display improvements in metabolic parameters (Supplementary Fig. 5). Instead, MKO mice exhibited a selective reduction in adipose Treg cells without affecting Th1 responses (Supplementary Fig. 6), suggesting that macrophage-derived OX40L may preferentially regulate Treg homeostasis rather than driving obesity-associated metabolic dysfunction. In contrast, adipocyte-specific OX40L deletion markedly reduced adipose Th1 accumulation and improved insulin sensitivity (Fig. 3 and Fig. 4), and restoration of Th1 cells reversed this metabolic protection (Fig. 5). Mechanistically, adipocyte-derived OX40L promoted Th1 proliferation and differentiation but did not affect the effector function of mature Th1 cells (Fig. 6 and Supplementary Fig. 12). Dendritic cells (DCs) are the most potent professional APCs and have been reported to contribute to obesity-associated inflammation and insulin resistance, in part through their APC function^43,44^. To evaluate whether DCs contribute to OX40L-mediated Th1 responses in adipose tissue, we first analysed a publicly available dataset of adipose tissue DCs (GSE85846). This analysis revealed no significant difference in OX40L expression between ND and HFD conditions (Supplementary Fig. 14a). Moreover, in vitro co-culture experiments using bone marrow-derived dendritic cells and naïve CD4⁺ T cells showed that OX40L blockade did not affect DC-mediated Th1 differentiation or IFNγ production (Supplementary Fig. 14b, c). These findings suggest that, although DCs are classical professional APCs, DC-derived OX40L is unlikely to play a dominant role in driving Th1 responses during obesity. Collectively, these findings identify adipocyte-derived OX40L as a key regulator of Th1-driven adipose inflammation and insulin resistance, highlighting its potential as a therapeutic target for metabolic disease.

The OX40–OX40L axis has emerged as a promising therapeutic target in T cell-mediated diseases^45^. Antibody-mediated blockade of OX40 or OX40L has shown efficacy in multiple autoimmune models, including experimental autoimmune encephalomyelitis^46,47^, systemic lupus erythematosus^48^, rheumatoid arthritis^49,50^ and inflammatory bowel disease^51–53^. In addition, clinical trials targeting the OX40/OX40L co-stimulatory pathway have demonstrated therapeutic efficacy in atopic dermatitis^54,55^. Notably, Amgen’s rocatinlimab (an anti-OX40 monoclonal antibody) and Sanofi’s amlitelimab (an anti-OX40L monoclonal antibody), two novel biologics disrupting T cell activation via the OX40/OX40L axis, have shown durable clinical responses in patients with atopic dermatitis^56–58^. Our findings extend this paradigm to metabolic disease, demonstrating that treatment with an OX40L-blocking antibody reduces Th1/Th17 dominance and increases Th2 and Treg populations in eWAT and improves insulin sensitivity in obese mice (Fig. 7). Importantly, systemic inhibition of the OX40 pathway has not been associated with serious side effects in clinical trials^45^. However, given the pleiotropic functions of OX40 in T cell biology, selectively targeting adipocyte-derived OX40L represents a more precise therapeutic strategy to mitigate adipose inflammation without compromising systemic immune function. To this end, we propose the development of adipocyte-targeted delivery systems. Innovative platforms with adipocyte specificity could enable adipose-restricted OX40L blockade, thereby conferring anti-inflammatory effects that are localized to adipose depots while preserving systemic immune competence. Such spatially targeted approaches may address a critical unmet need in the development of tissue-specific immunotherapies for obesity-related metabolic dysfunction.

Previous studies have examined the response of global OX40-knockout (*OX40*^*−/−*^) mice to HFD challenge^59^. However, to our knowledge, the present study is the first to provide an in-depth investigation of the role of adipose OX40L in obesity-induced adipose inflammation and insulin resistance. Liu et al. reported that *OX40*^*−/−*^ mice fed a HFD exhibited reduced weight gain along with improved adipose inflammation and insulin resistance compared with WT mice^59^. As weight loss is inherently linked to improvements in adipose inflammation and insulin resistance, their study could not rule out the possibility that these metabolic benefits were secondary to reduced weight gain. In contrast, we observed no significant differences in HFD-induced weight gain between *OX40*^*−/−*^, AKO or MKO mice and their respective WT controls. Notably, Liu et al. purchased age-matched male WT and *OX40*^*−/−*^ mice from The Jackon Laboratory, and mice of different genotypes were probably housed separately. In our study, by contrast, WT and *OX40*^*−/−*^ mice were littermates generated through heterozygous breeding and were co-housed. The discrepancy in HFD-induced weight gain between our findings and those of Liu et al. may be attributed to differences in breeding and housing conditions, as well as potential variations in microbiome composition. Thus, the current study provides a comprehensive analysis using multiple independent measures to establish a direct role of adipocyte-derived OX40L in obesity-induced Th1 activation in adipose tissue and insulin resistance.

**Fig. 8:**
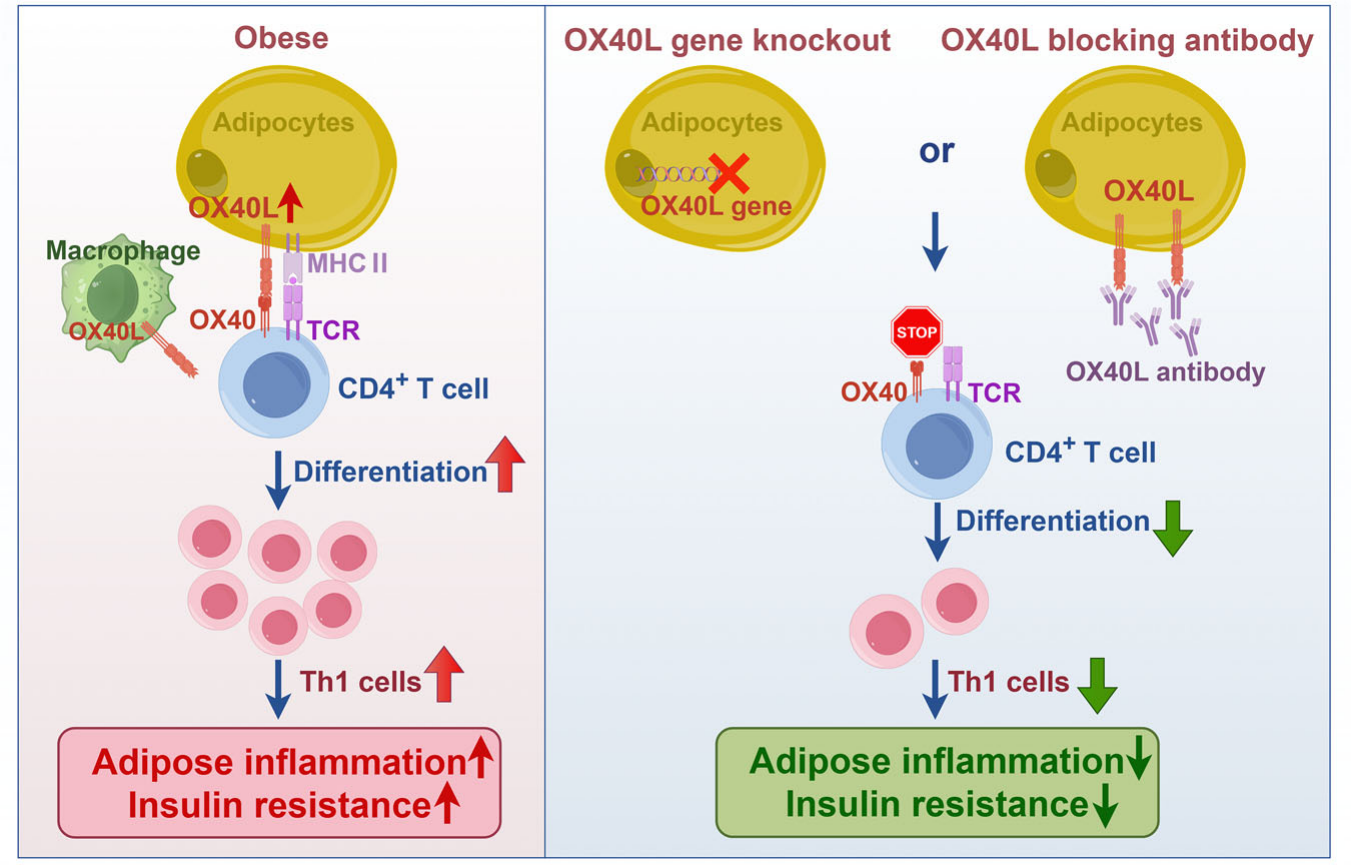
The role of adipocyte OX40L in obesity-induced adipose inflammation and insulin resistance. In the context of obesity, OX40L expression is notably upregulated in both adipocytes and macrophages. However, it is the adipocyte-specific expression of OX40L, rather than macrophage-derived OX40L, that promotes the differentiation of CD4^+^ T cells into Th1 cells. These Th1 cells accumulate in the visceral adipose tissue, thereby exacerbating local inflammation and contributing to insulin resistance. In contrast, genetic ablation of OX40L in adipocytes markedly reduces Th1 cell infiltration, alleviates inflammatory responses and enhances insulin sensitivity, all without influencing adiposity. Moreover, therapeutic inhibition of OX40L via neutralizing antibodies recapitulates these beneficial effects, providing further evidence that OX40L is a promising target for immunotherapeutic strategies in metabolic disorders. This figure was created in Figdraw.

The results presented here clearly demonstrate that adipocyte OX40L plays a pivotal role in promoting Th1 accumulation and IFNγ production in HFD-fed mice, thereby driving adipose inflammation and systemic insulin resistance. Disruption of this signalling, either through reduced adipocyte OX40L expression or administration of an OX40L-blocking antibody, ameliorates adipose inflammation and preserves systemic insulin sensitivity (Fig. 8). These findings position adipose OX40L-OX40 signalling as a potential therapeutic target for mitigating obesity-associated metabolic dysfunction.

## Supplementary information

Supplementary Information

Source data
