# Supplementary material for: Adipocyte OX40L promotes adipose T cell activation and insulin resistance in obesity
Source: Exp Mol Med. 2026 Jul 6;58(7):2293–307. doi: 10.1038/s12276-026-01770-8 (PMC13434776; doi:10.1038/s12276-026-01770-8)
Supplement: Supplementary file 1 [file 12276_2026_1770_MOESM1_ESM.docx]

**Supplementary Fig. 1: Obesity induces OX40L expression in adipocytes and ATMs.** (**a**) RT-qPCR analysis of mRNA expression of various costimulatory molecules in eWAT adipocytes from 12-weeks ND and HFD mice (n = 6–7 per group). (**b**) RT-qPCR analysis of mRNA expression of various costimulatory molecules in eWAT macrophages from 12-weeks ND and HFD mice (n = 3–6 per group). (**c**) RT-qPCR analysis of OX40L mRNA expression in eWAT adipocytes from WT and *ob/ob* mice (n = 3 per group). (**d**) RT-qPCR analysis of OX40L mRNA expression in eWAT macrophages from WT and *ob/ob* mice (n = 3 per group). (**e**) RT-qPCR analysis of OX40L mRNA expression in various tissues from 12-weeks ND and HFD mice (n = 6 per group). (**f**) Western blot analysis of OX40L protein expression in eWAT, eWAT adipocytes and ATMs from 12-weeks ND and HFD mice (n = 2 per group). (**g**) Flow cytometry analysis of OX40L⁺ cells among adipocytes and ATMs from ND- and HFD-fed mice, in this experiment, adipocytes and ATMs were stained in the same tube (n = 3 per group). (**h**) Mean fluorescence intensity (MFI) of OX40L in adipocytes and ATMs under the same conditions (n = 3 per group). (**i**) RT-qPCR analysis of OX40L mRNA expression in human VAT adipocytes (n = 10–16 per group). Data represent mean ± SEM. ^+^*P* < 0.1, **P* < 0.05, ***P* < 0.01, ****P* < 0.001, and *****P* < 0.0001.

 **Supplementary Fig. 2: Characterization of adipocytes by flow cytometry.** (**a**) Adipocyte flow gating strategy. (**b**) Bright-field images of adipocytes following flow cytometry staining (scale bar: 50 μm). (**c**) Representative fluorescence images of adipocytes stained for OX40L (scale bar: 20 μm).

**Supplementary Fig. 3: Single-nucleus RNA-seq analysis of OX40L expression across different cell types in human and mouse visceral adipose tissue.** (**a**) UMAP visualization showing major cell populations identified in single-nucleus RNA-seq datasets from mouse perigonadal adipose tissue (PG) under chow diet (Chow_PG) and high-fat diet (HFD_PG) conditions. (**b**) Feature plots showing OX40L expression in mouse adipose tissue cell populations under Chow_PG and HFD_PG conditions. (**c**) Quantification of the percentage of OX40L-positive cells across different cell types in mouse adipose tissue under Chow_PG and HFD_PG conditions. (**d**) UMAP visualization showing major cell populations identified in single-nucleus RNA-seq datasets from human visceral adipose tissue from lean (Lean_VAT) and obese (Obese_VAT) individuals. (**e**) Feature plots showing OX40L expression across cell populations in human VAT from lean and obese subjects. (**f**) Quantification of the percentage of OX40L-positive cells across different cell types in human VAT under lean and obese conditions.

**Supplementary Fig. 4: OX40 deficiency alters CD4⁺ T cell subsets in eWAT.** (**a**) Macrophage flow gating strategy. (**b**) T cell flow gating strategy. (**c**) Flow cytometry analysis of Th2, Th17 and Treg cell populations in eWAT from *OX40*^−/−^ and WT mice (n = 6 per group). (**d**) Number of Th2, Th17 and Treg cell per gram of eWAT (n = 6 per group). Data represent mean ± SEM. **P* < 0.05, ***P* < 0.01, and ****P* < 0.001.

**Supplementary Fig. 5: Macrophage-specific OX40L deficiency does not affect HFD-induced insulin resistance.** Macrophage-specific OX40L knockout (MKO) and littermate control (MWT) mice were fed a HFD for 12 weeks. (**a**) RT-qPCR analysis of OX40L mRNA expression in eWAT ATMs, BMDMs, and eWAT adipocytes from MKO and MWT mice (n = 4 per group). (**b**) Flow cytometry analysis of OX40L^+^ cell proportions in eWAT macrophages from MKO and MWT mice (n = 5 per group). (**c**) Body weight of MKO and MWT mice (n = 5 per group). (**d**) Representative images of MKO and MWT mice. (**e**) Representative images of BAT, iWAT, eWAT, and liver from MKO and MWT mice. (**f**) Weight percentages of BAT, iWAT, eWAT, and liver relative to body weight (n = 5 per group). (**g**) ITT results for MKO and MWT mice (n = 5 per group). (**h**) GTT results for MKO and MWT mice (n = 5 per group). (**i**) FPG levels of MKO and MWT mice (n = 5 per group). (**j**) FPI levels of MKO and MWT mice (n = 5 per group). Data represent mean ± SEM. **P* < 0.05, ***P* < 0.01, and *****P* < 0.0001.

**Supplementary Fig. 6: Macrophage-specific OX40L deficiency reduces Treg cells in visceral adipose tissue.** (**a**) Flow cytometry analysis of CD4^+^ and CD8^+^ T cell populations in eWAT and spleen from MKO and MWT mice (n = 6 per group). (**b**) Flow cytometry analysis of Th1 cell populations in eWAT and spleen from MKO and MWT mice (n = 6 per group). (**c**) Flow cytometry analysis of Th2 cell populations in eWAT and spleen from MKO and MWT mice (n = 6 per group). (**d**) Flow cytometry analysis of Th17 cell populations in eWAT and spleen from MKO and MWT mice (n = 6 per group). (**e**) Flow cytometry analysis of Treg cell populations in eWAT and spleen from MKO and MWT mice (n = 6 per group). Data represent mean ± SEM. ***P* < 0.01.

 **Supplementary Fig. 7: The establishment of adipocyte-specific OX40L knockout mice model.** AKO and AWT mice were fed a HFD for 12 weeks. (**a**) RT-qPCR analysis of OX40L mRNA expression in various tissues from AKO and AWT mice (n = 5 per group). (**b**) RT-qPCR analysis of OX40L mRNA expression in eWAT adipocytes, SVF, and BMDM from AKO and AWT mice (n = 4 per group). (**c**) Western blot analysis of OX40L protein expression in eWAT adipocytes and SVF from AKO and AWT mice (n = 3 per group). (**d**) Flow cytometry analysis of OX40L^+^ cell proportions in eWAT adipocytes from AKO and AWT mice (n = 4 per group). (**e**) ELISA quantification of IFNγ levels in eWAT culture supernatants from HFD-fed AKO and AWT mice (n = 3 per group). (**f**) ELISA quantification of TNFα levels in eWAT culture supernatants from HFD-fed AKO and AWT mice (n = 3 per group). Data represent mean ± SEM. **P* < 0.05, ***P* < 0.01, and ****P* < 0.001.

**Supplementary Fig. 8:** **Adipocyte-specific OX40L deficiency has no effect on insulin sensitivity and T cell accumulation in adipose tissue under ND-fed condition.** AKO and AWT mice were fed a ND for 12 weeks. (**a**) Body weight of AKO and AWT mice (n = 7–8 per group). (**b**) ITT results for AKO and AWT mice (n = 7–8 per group). (**c**) GTT results for AKO and AWT mice (n = 7–8 per group). (**d**) FPG levels of AKO and AWT mice (n = 7–8 per group). (**e**) FPI levels of AKO and AWT mice (n = 5–8 per group). (**f**) Flow cytometry analysis of CD4^+^ and CD8^+^ T cell populations in eWAT and spleen from AKO and AWT mice (n = 4–5 per group). (**g**) Flow cytometry analysis of Th1 cell populations in eWAT and spleen from AKO and AWT mice (n = 4–5 per group). (**h**) Flow cytometry analysis of Th2 cell populations in eWAT and spleen from AKO and AWT mice (n = 4–5 per group). (**i**) Flow cytometry analysis of Th17 cell populations in eWAT and spleen from AKO and AWT mice (n = 4–5 per group). (**j**) Flow cytometry analysis of Treg cell populations in eWAT and spleen from AKO and AWT mice (n = 4–5 per group). Data represent mean ± SEM.

**Supplementary Fig. 9:** **Adipocyte-specific OX40L deficiency reduces lipid accumulation in liver and skeletal muscle.** (**a**) Representative H&E and Oil Red O staining of liver and skeletal muscle sections from HFD-fed AWT and AKO mice, Scale bar: 100 μm. RT-qPCR analysis of genes involved in fatty acid synthesis, fatty acid uptake, and lipid droplet formation in the gastrocnemius muscle (**b**) and liver (**c**) of HFD-fed AWT and AKO mice (n = 5–6 per group). Immunohistochemical staining of OX40L in the gastrocnemius muscle (**d**) and liver (**e**) of HFD-fed AWT and AKO mice and semiquantitative analysis of OX40L expression, Scale bar: 100 μm, AOD: Average optical density (n = 6 per group). Data represent mean ± SEM. ^+^*P* < 0.1, **P* < 0.05 and ***P* < 0.01.

**Supplementary Fig. 10: Adipocyte-specific OX40L deletion does not affect CD8⁺ T cell effector function in adipose tissue.** (**a**) Flow cytometry analysis of IFNγ⁺ CD8⁺ T cells proportions in eWAT from HFD-fed AKO and AWT mice (n = 6 per group). (**b**) Mean fluorescence intensity (MFI) of IFNγ in CD8⁺ T cells (n = 6 per group). (**c**) Flow cytometry analysis of FasL^+^ cell populations among CD8⁺ T cells in eWAT from HFD-fed AKO and AWT mice (n = 6 per group). (**d**) MFI of FasL in CD8⁺ T cells (n = 6 per group). (**e**) Flow cytometry analysis of IFNγ⁺ CD8⁺ T cells proportions after coculture of adipocytes isolated from HFD-fed AWT or AKO mice with purified CD8⁺ T cells (n = 3 per group). (**f**) Flow cytometry analysis of GrazB⁺ CD8⁺ T cells proportions under the same coculture conditions (n = 3 per group). (**g**) Flow cytometry analysis of Perforin⁺ CD8⁺ T cells proportions under the same coculture conditions (n = 3 per group). (**h**) ELISA quantification of IFNγ levels under the same coculture conditions (n = 3 per group). Data represent mean ± SEM.

**Supplementary Fig. 11:** **Adoptively transferred Th1 cells preferentially traffic to adipose tissue and accumulate in AKO mice.** (**a**) Flow cytometry analysis of CD45.1^+^ cell populations in eWAT and spleen from AKO and AWT mice after Th1 cells injection (n = 5–6 per group). (**b**) Number of CD45.1^+^ Th1 cells per gram of eWAT (n = 5–6 per group). Data represent mean ± SEM. ^+^*P* < 0.1 and *****P* < 0.0001.

**Supplementary Fig. 12:** **Adipocyte-derived OX40L promotes Th1 differentiation but does not affect the effector function of differentiated Th1 cells.** (**a**) Flow cytometry analysis of Th1 cell proportions after coculture of adipocytes isolated from HFD-fed AWT mice with naïve CD4⁺ T cells from OT-II mice in the presence or absence of OVA, together with either α-OX40L blocking antibody or IgG2b antibody (n = 3 per group). (**b**) ELISA quantification of IFNγ levels in coculture supernatants (n = 3 per group). (**c**) Flow cytometry analysis of Th1 cell proportions after coculture of adipocytes isolated from HFD-fed AWT or AKO mice with in vitro differentiated Th1 cells (n = 3 per group). (**d**) MFI of IFNγ in Th1 cells under the conditions shown in (**c**) (n = 3 per group). (**e**) ELISA quantification of IFNγ levels under the conditions shown in (**c**) (n = 3 per group). (**f**) Flow cytometry analysis of Th1 cell proportions after coculture of adipocytes isolated from HFD-fed AWT mice with in vitro differentiated Th1 cells with either α-OX40L blocking antibody or IgG2b antibody (n = 3 per group). (**g**) MFI of IFNγ in Th1 cells under the conditions shown in (**f**) (n = 3 per group). (**h**) ELISA quantification of IFNγ levels under the conditions shown in (**f**) (n = 3 per group). Data represent mean ± SEM. **P* < 0.05, ***P* < 0.01, ****P* < 0.001 and *****P* < 0.0001.

**Supplementary Fig. 13:** **OX40L blocking antibody** **does not alter body composition, and** **baseline metabolic profiles showed no intergroup differences.** (**a**) Representative images of α-OX40L and IgG2b-treated mice and their BAT, iWAT, eWAT, and liver. (**b**) Weight percentages of BAT, iWAT, eWAT, and liver relative to body weight (n = 7–8 per group). (**c**) ITT results before antibody treatment (n = 7–8 per group). (**d**) GTT results before antibody treatment (n = 7–8 per group). (**e**) FPG levels after α-OX40L or IgG2b treatment 4 weeks (n = 6–8 per group). (**f**) FPI levels after α-OX40L or IgG2b treatment 4 weeks (n = 5–6 per group). Data represent mean ± SEM. **P* < 0.05.

**Supplementary Fig. 14: Dendritic cell derived OX40L does not contribute to Th1 differentiation.** (**a**) Analysis of a publicly available microarray dataset (GSE85846) showing OX40L gene expression in adipose tissue derived dendritic cells isolated from ND and HFD mice. Bone marrow-derived dendritic cells coculture with naïve CD4⁺ T cells from OT-II mice in the presence or absence of OVA stimulation and α-OX40L antibody or IgG2b antibody. (**b**) Flow cytometry analysis of Th1 cell proportions in coculture systems (n = 3 per group). (**c**) ELISA quantification of IFNγ levels in coculture supernatants (n = 3 per group). Data represent mean ± SEM. ****P* < 0.001 and *****P* < 0.0001.

**Supplementary Table 1: mRNA primer sequences (m: mouse; h: human)**

| **Genes** | **Primers (5’-3')** |
| --- | --- |
| *m36b4* | Forward: TTATAACCCTGAAGTGCTCGAC |
|  | Reverse: GTAAGTGGGAAGGTGTACTCAG |
| *mβ-actin* | Forward: ACCGTGAAAAGATGACCCAG |
|  | Reverse: GAGCATAGCCCTCGTAGATG |
| *mAdiponectin* | Forward: TGTTCCTCTTAATCCTGCCCA |
|  | Reverse: CCAACCTGCACAAGTTCCCTT |
| *mArg1* | Forward: CTCCAAGCCAAAGTCCTTAGAG |
|  | Reverse: AGGAGCTGTCATTAGGGACATC |
| *mCd3* | Forward: CTCAGAAGCATGATAAGCAC |
|  | Reverse: CAGAGTGATACAGATGTCAAC |
| *mCd4* | Forward: GTTCGGCATGACACTCTCAG |
|  | Reverse: CCTTCTCTGCCTTCCACATC |
| *mCd8* | Forward: AGTTAACAAGCATCTACTGGCT |
|  | Reverse: TCTTGTCCACACTTTCACCATA |
| *mCd11b* | Forward: CATCCCATGACCTTCCAAGAG |
|  | Reverse: GACATATTCACAGCCTCTGGAG |
| *mCd40* | Forward: ACAAACAGTACCTCCACGATG |
|  | Reverse: TTCTTAACCCGAAGCCCTTG |
| *mCd48* | Forward: GAGGCTATCGTGTGAGGTAAAG |
|  | Reverse: GCAGTCCAACATACTCCAGAAG |
| *mCd70* | Forward: CTGTTGGTTTCATTGTAGCGG |
|  | Reverse: CTGGATATGCAGCCTGTAGAG |
| *mCd80* | Forward: CAACTGTCCAAGTCAGTGAAAG |
|  | Reverse: CACCACTTTGTCATGTTTTTGC |
| *mCd86* | Forward: ACGGAGTCAATGAAGATTTCCT |
|  | Reverse: GATTCGGCTTCTTGTGACATAC |
| *mCd206* | Forward: ATGGATGTTGATGGCTACTGG |
|  | Reverse: TTCTGACTCTGGACACTTGC |
| *mCiita* | Forward: GATCCTTCCAGCCTTCTCTTC |
|  | Reverse: GTTTTGGGACAAGTTGAGCG |
| *mEmr1* | Forward: TTTCAAATGGATCCAGAAGG |
|  | Reverse: CAGAAGGAAGCATAACCAAG |
| *mFoxp3* | Forward: TTTCACCTATGCCACCCTTATC |
|  | Reverse: CATGCGAGTAAACCAATGGTAG |
| *mGata3* | Forward: ATTACCACCTATCCGCCCTAT |
|  | Reverse: CGGTTCTGCCCATTCATTTTAT |
| *mH2ab1* | Forward: TGCCATTACCTGTGCCTTAG |
|  | Reverse: CCTCCCGGTTGTAGATGTATC |
| *mH2eb1* | Forward: ATTACCACCTATCCGCCCTAT |
|  | Reverse: CGGTTCTGCCCATTCATTTTAT |
| *mIFNγ* | Forward: CTTTGGACCCTCTGACTTGAG |
|  | Reverse: TCTTCCACATCTATGCCACTTG |
| *mIL-Iβ* | Forward: GGTCAAAGGTTTGGAAGCAG |
|  | Reverse: TGTGAAATGCCACCTTTTGA |
| *mIL-6* | Forward: AGACTTCCATCCAGTTGCCTT |
|  | Reverse: TTCTCATTTCCACGATTTCCC |
| *mIL-12* | Forward: CTCCTGGTTTGCCATCGTTT |
|  | Reverse: GGGAGTCCAGTCCACCTCTA |
| *mIL-17a* | Forward: GACTACCTCAACCGTTCCAC |
|  | Reverse: TTCTCGACCCTGAAAGTGAAG |
| *mLeptin* | Forward: GTGCCTATCCAGAAAGTCCAG |
|  | Reverse: GACCTGTTGATAGACTGCCAG |
| *mMCP1* | Forward: AGGTCCCTGTCATGCTTCTG |
|  | Reverse: TCTGGACCCATTCCTTCTTG |
| *mMrc1* | Forward: CCTATGAAAATTGGGCTTACGG |
|  | Reverse: CTGACAAATCCAGTTGTTGAGG |
| *mNlrp3* | Forward: CTCCAACCATTCTCTGACCAG |
|  | Reverse: ACAGATTGAAGTAAGGCCGG |
| *mPVR* | Forward: TTTGTCCTCGCTGTAGTCATC |
|  | Reverse: GAGTAGGGTTCCAGTTGCTATG |
| *mslamf1* | Forward: AAAACCTGAGCCTGAAGATCC |
|  | Reverse: AGCTGTAAGTCACATGGTCC |
| *mTbet* | Forward: GATCACTCAGCTGAAAATCGAC |
|  | Reverse: AGGCTGTGAGATCATATCCTTG |
| *mTimd4* | Forward: ATTCTCCCATCCACTTCACAG |
|  | Reverse: GGACGCTGTCACTATCTTCAG |
| *mTnfα* | Forward: TACTTAGACTTTGCGGAG |
|  | Reverse: AGAGTAAAGGGGTCAGAG |
| *mTnfsf4* | Forward: TGCTTCATCTATGTCTGCCTG |
|  | Reverse: CAACCGAATTGTTCTGCACC |
| *mTnfsf8* | Forward: GCGATCATTCTGGTACTGGTAG |
|  | Reverse: AATTGGACTATCAGGTTCCCG |
| *mTnfsf9* | Forward: GCGATCATTCTGGTACTGGTAG |
|  | Reverse: AATTGGACTATCAGGTTCCCG |
| *mTnfrsf14* | Forward: AGGGCAGAGGGTAGAGAAG |
|  | Reverse: GCAAAAGGATGGACACAACG |
| *mTnfsf14* | Forward: GGTTTCTCCTGAGACTGCATC |
|  | Reverse: GATACGTCAAGCCCCTCAAG |
| *mTnfsf15* | Forward: TCATTTCCCATCCTCGCAG |
|  | Reverse: GTCATGTTCCCAGTGTAGAGC |
| *mTnfsf18* | Forward: CCCCAGTTCTCATTCCATCAG |
|  | Reverse: AGGTTTGGGAGATGTCATGTG |
| *mYm1* | Forward: GAAGGAGCCACTGAGGTCTG |
|  | Reverse: GAGCCACTGAGCCTTCAAC |
| *mSrebp1c* | Forward: GGAGCCATGGATTGCACATT |
|  | Reverse: CCTGTCTCACCCCCAGCATA |
| *mFasn* | Forward: GGCATCATTGGGCACTCCTT |
|  | Reverse: GCTGCAAGCACAGCCTCTCT |
| *mACC* | Forward: GCCATTGGTATTGGGGCTTAC |
|  | Reverse: CCCGACCAAGGACTTTGTTG |
| *mScd1* | Forward: CTGACCTGAAAGCCGAGAAG |
|  | Reverse: AGAAGGTGCTAACGAACAGG |
| *mCd36* | Forward: GCGACATGATTAATGGCACAG |
|  | Reverse: GATCCGAACACAGCGTAGATAG |
| *mFabp4* | Forward: AAGGTGAAGAGCATCATAACCCT |
|  | Reverse: TCACGCCTTTCATAACACATTCC |
| *mFabp5* | Forward: GGAGAGCACGATAACAAGAAAAC |
|  | Reverse: AGCATATTCACTCTGGCAGC |
| *mPLIN1* | Forward: ACAGACACAGAGGGAGAGG |
|  | Reverse: AGTGTTCTGCACGGTGTG |
| *mPLIN4* | Forward: AAACAGCAACAGACCCCTC |
|  | Reverse: AACTTCCCATGTCCTTGTCTC |
| *mCAV1* | Forward: TTCTCTTAAATCACAGCCCAGG |
|  | Reverse: TGTAGATGTTGCCCTGTTCC |
| *mCavin1* | Forward: AAGAAACTGGAGGTCAACGAG |
|  | Reverse: CCGACTCTTTCAGCGACTTG |
| *h36B4* | Forward: CAGCAAGTGGGAAGGTGTAATCC |
|  | Reverse: CCCATTCTATCATCAACGGGTACAA |
| *hTNFSF4* | Forward: TCGAGAGGAACAAGCTATTGC |
|  | Reverse: TTGGGAAGTGAGGATGAAACC |

**Supplementary Table 2: Phenotypic parameters of the nonobese (BMI < 30 kg/m^2^) and obese (BMI ≥ 30 kg/m^2^) human donors.**

| **Subject** | **Age**  **(years)** | **Gender** | **BMI**  **(kg/m^2^)** | **FPG**  **(mmol/L)** | **FPI**  **(mU/mL)** | **Cholesterol (mg/dL)** | **Triglyceride (mg/dL)** | **HDL (mg/dL)** | **LDL (mg/dL)** |
| --- | --- | --- | --- | --- | --- | --- | --- | --- | --- |
| Lean 1 | 53 | Female | 23.7 | 4.33 | 2.02 | 146 | 101 | 45 | 84 |
| Lean 2 | 34 | Female | 20.4 | 4.06 | 5.64 | 197 | 96 | 72 | 96 |
| Lean 3 | 70 | Female | 19.8 | 4.39 | 4.47 | 174 | 197 | 51 | 78 |
| Lean 4 | 53 | Female | 16.3 | 4.11 | 5.04 | 142 | 63 | 40 | 81 |
| Lean 5 | 65 | Female | 19.2 | 3.50 | 5.63 | 183 | 55 | 73 | 81 |
| Lean 6 | 49 | Female | 20.9 | 4.22 | 6.82 | 145 | 86 | 55 | 71 |
| Lean 7 | 56 | Female | 23.3 | 4.28 | 2.52 | 131 | 198 | 33 | 56 |
| Lean 8 | 55 | Female | 22.3 | 4.83 | 4.56 | 182 | 64 | 65 | 91 |
| Lean 9 | 78 | Female | 27.2 | 4.83 | 5.60 | 191 | 158 | 59 | 94 |
| Lean 10 | 58 | Female | 25.0 | 6.22 | 2.16 | 163 | 116 | 49 | 84 |
| Obese 1 | 72 | Female | 31.8 | 4.22 | 3.16 | 213 | 143 | 56 | 144 |
| Obese 2 | 54 | Female | 44.2 | 4.17 | 6.53 | 195 | 69 | 80 | 101 |
| Obese 3 | 45 | Female | 33.6 | 4.11 | 5.96 | 150 | 75 | 46 | 82 |
| Obese 4 | 51 | Female | 31.9 | 6.50 | 5.06 | 206 | 73 | 63 | 127 |
| Obese 5 | 59 | Female | 30.3 | 4.67 | 3.03 | 171 | 79 | 57 | 107 |
| Obese 6 | 72 | Female | 30.5 | 5.06 | 3.91 | 205 | 128 | 38 | 132 |
| Obese 7 | 50 | Female | 47.3 | 8.06 | 11.14 | 263 | 310 | 40 | 184 |
| Obese 8 | 45 | Female | 47.6 | 6.67 | 6.97 | 203 | 123 | 64 | 116 |
| Obese 9 | 42 | Female | 36.5 | 9.50 | 3.54 | 226 | 157 | 52 | 166 |
| Obese 10 | 55 | Female | 37.5 | 3.78 | 2.03 | 231 | 124 | 42 | 151 |
| Obese 11 | 63 | Female | 31.8 | 4.17 | 4.10 | 257 | 82 | 61 | 148 |
| Obese 12 | 65 | Female | 38.0 | 6.17 | 2.54 | 284 | 273 | 34 | 175 |
| Obese 13 | 64 | Female | 35.0 | 4.33 | 5.18 | 216 | 94 | 56 | 139 |
| Obese 14 | 46 | Female | 41.2 | 4.33 | 2.08 | 292 | 270 | 61 | 142 |
| Obese 15 | 56 | Female | 39.9 | 4.89 | 2.22 | 196 | 112 | 54 | 121 |
| Obese 16 | 58 | Female | 33.4 | 5.11 | 2.15 | 222 | 257 | 61 | 129 |

Phenotype abbreviations: body mass index (BMI), fasting plasma glucose (FPG), fasting plasma insulin (FPI), high-density lipoprotein cholesterol (HDL) and low-density lipoprotein cholesterol (LDL).
